# Responses of maize roots, rhizosphere enzyme kinetics and prokaryote diversity to alternating precipitation: insights from a three-year field study

**DOI:** 10.1093/aob/mcaf180

**Published:** 2025-08-06

**Authors:** Henrike Würsig, Bunlong Yim, María Martín Roldán, Negar Ghaderi, Florian Stoll, Marie-Lara Bouffaud, Doris Vetterlein, Thomas Reitz, Evgenia Blagodatskaya, Kornelia Smalla, Mika Tarkka

**Affiliations:** Department of Soil Ecology, Helmholtz Centre for Environmental Research, Halle 06120, Germany; Institute for Epidemiology and Pathogen Diagnostics, Julius Kühn Institute, Braunschweig 38104, Germany; Department of Soil Ecology, Helmholtz Centre for Environmental Research, Halle 06120, Germany; Department of Soil Ecology, Helmholtz Centre for Environmental Research, Halle 06120, Germany; Institute of Terrestrial Ecosystems,Federal Institute of Technology, Zurich 8092, Switzerland; Department of Soil Ecology, Helmholtz Centre for Environmental Research, Halle 06120, Germany; Department of Soil Ecology, Helmholtz Centre for Environmental Research, Halle 06120, Germany; Institute of Agricultural and Nutritional Sciences, Martin-Luther-University Halle-Wittenberg, Halle 06120, Germany; Department of Soil Ecology, Helmholtz Centre for Environmental Research, Halle 06120, Germany; German Centre for Integrative Biodiversity Research (iDiv) Halle-Jena Leipzig, Leipzig 04103, Germany; Department of Soil Ecology, Helmholtz Centre for Environmental Research, Halle 06120, Germany; Institute for Epidemiology and Pathogen Diagnostics, Julius Kühn Institute, Braunschweig 38104, Germany; Department of Soil Ecology, Helmholtz Centre for Environmental Research, Halle 06120, Germany; German Centre for Integrative Biodiversity Research (iDiv) Halle-Jena Leipzig, Leipzig 04103, Germany

**Keywords:** ACC deaminase, agriculture, bacteria, drought, enzyme activity, field, gene expression, monoculture, rhizosphere, root, root hair, *Zea mays*

## Abstract

**Background:**

Understanding how annual weather variation, including droughts, affects plant roots and rhizosphere prokaryote dynamics in different years is essential for predicting plant responses to climate fluctuations. This study aimed to investigate the effects of alternating dry and moist years on maize root gene expression and rhizosphere prokaryote composition, and to reveal interactions between the two.

**Methods:**

*Zea mays* B73 wild-type (WT) and a root hair-deficient mutant (*rth3*) were grown on two substrates during a 3-year field experiment with alternating precipitation, designated as dry, moist and dry. Root gene expression was analysed between the two dry years and the moist year, supported by superoxide dismutase activity. The rhizosphere was analysed by measuring the enzyme kinetic parameters β-glucosidase, acid phosphatase, leucine aminopeptidase and *N*-acetylglucosaminidase, accompanied by the 16S rRNA-based and 1-aminocyclopropane-1-carboxylate deaminase (*acdS*^+^)-based microbial community.

**Key Results:**

Year was the main driver of root gene expression and the 16S rRNA-based microbial community, with a distinct pattern of drought-responsive genes between dry years and the moist year. Substrate was the main driver of the *acdS*^+^*-*based microbial community and influenced root gene expression and the 16S rRNA-based microbial community, indicating interactive effects between maize roots and rhizosphere prokaryotes. The effect of year and substrate on enzyme kinetics was enzyme-specific. Root hair presence had a marginal effect.

**Conclusions:**

This study highlights the role of annual weather variation in shaping root gene expression, rhizosphere prokaryotes and enzyme kinetics and underlines the role of substrate in structuring *acdS*^+^*-*based microbial communities. Our results suggest that plant–microbe interactions are highly sensitive to precipitation variability and might be influenced by repeated maize planting. They emphasize the importance of precipitation history in shaping plant–microbe interactions, which can serve as a basis for drought resilience strategies in agriculture.

## INTRODUCTION

Drought is one of the most challenging problems affecting plant growth and crop productivity (FAO, 2023). Maize, for example, which makes a substantial contribution to agriculture in Europe, with annual yields of 103 million tonnes, can suffer yield losses of up to 40 % due to drought (Daryanto *et al*., 2016; FAO, 2022). In recent years, drought events even increased in intensity and duration (UBA, 2023), making an understanding of the processes involved particularly important to ensure successful crop production in the future. Being the first organ to sense a decrease in soil water availability, the plant root system plays a central role in sensing and transmitting the water status to the above-ground parts of the plant (Schachtman and Goodger, 2008; Kang *et al*., 2022). While the central role of root system architecture in drought tolerance is well established, the relative importance of root hairs and rhizosphere microbiomes in drought acclimation in the field is poorly understood (Marin *et al*., 2021; Ali *et al*., 2022).

Lack of plant-available water in soil leads to a variety of morphological, physiological and metabolic changes in roots, the severity of which depends on the plant growth stage (Song *et al*., 2019; Liu *et al*., 2021; Singh *et al*., 2023), and drought duration. In general, restricted root growth due to lack of photosynthates, which is indicative of severe drought stress (Körner, 2015), is contrasted by increased root length and root hair density, and extensive and branched root systems during moderate drought (Kang *et al*., 2022; Peer *et al*., 2024). Plant responses to moderate drought often improve water and nutrient uptake efficiency (Seleiman *et al*., 2021), and lead to increased root to shoot ratio (Jiang *et al*., 2012).

Drought triggers the accumulation of reactive oxygen species (ROS) such as hydrogen peroxide (H_2_O_2_) or superoxide radical (O_2_^−^), which activate stress-related signalling and cause protein damage and lipid peroxidation (Kang *et al*., 2022). Plants produce antioxidant enzymes to reduce oxidative stress, including superoxide dismutase (SOD), catalase, ascorbate peroxidase and glutathione reductase. SOD catalyses the reaction of the highly reactive O_2_^−^ into oxygen (O_2_) and the less reactive H_2_O_2_, which will be further reduced, among others, by catalase, ascorbate peroxidase and glutathione reductase (McCord and Fridovich, 1969; Karuppanapandian *et al*., 2011). After sensing the drought stress, roots accumulate the stress hormone abscisic acid (ABA), which has been shown to regulate the drought response in the whole plant body (Davies *et al*., 1986; O’Regan *et al*., 1993; Bahrun *et al*., 2002; Leach *et al*., 2011; Li *et al*., 2019). Apart from its function of regulating stomatal closure (Yan *et al*., 2017), an accumulation of ABA also ensures root growth by suppressing excess ethylene production and limiting cell stress (Saab *et al*., 1990; Sharp *et al*., 1994; Spollen *et al*., 2000). At the cell anatomical level, cell wall structure changes to facilitate root growth under moderate drought. For instance, expansins and xyloglucans can thereby loosen the cell walls and facilitate cell elongation under drought (Wu and Cosgrove, 2000; Kang *et al*., 2022). Another important mechanism of plant acclimatization to drought is the osmotic adjustment of the plant cytoplasm, whereby an accumulation of osmoprotectants such as proline, glycine betaine or soluble sugars helps to maintain cell turgor and protect cellular structures from dehydration. The responses described above are associated with differential gene expression. In maize, these responses lead to higher or lower expression of drought and heat stress marker genes, such as the definite sets of *aquaporin* water channels, *late embryogenesis abundant proteins*, *heat shock factors and -proteins*, and genes related to the biosynthesis and catabolism of ABA. Gene Ontology (GO) terms related to these genes also show an enrichment in up- or down-regulated genes in maize roots under water limitation (Opitz *et al*., 2014; Hao *et al*., 2020; Jiao *et al*., 2022; Kang *et al*., 2023).

Under drought, increased root exudation and altered root exudate composition can have a profound effect on the rhizosphere and the rhizosphere microbiome (Gargallo-Garriga *et al*., 2018; Canarini *et al*., 2019; Hartwig *et al*., 2025). The rhizosphere, which can be defined as the volume of soil influenced by plant roots (Hiltner, 1904), contains a wide variety of soil microorganisms, such as bacteria, fungi, archaea and protozoa (Mendes *et al*., 2013), which can be distinguished from the bulk soil, in terms of their diversity, abundance and composition (Bonkowski *et al*., 2021). The rhizosphere microbiome is thus influenced by soil properties, plant species and genotypes, and biotic and abiotic stressors (Berg and Smalla, 2009; van Dam and Bouwmeester, 2016; Yim *et al*., 2022, 2025). Through rhizodeposits, such as root exudates, plants can directly influence the composition and diversity of the rhizosphere microbiome (Berg and Smalla, 2009; Pieterse *et al*., 2014; Tiziani *et al*., 2022), and the rhizosphere microbiome can in turn influence root exudation (Korenblum *et al*., 2020). These changes in plant exudation patterns and rhizosphere microbiome composition will be reflected in rhizosphere enzyme kinetics. Extracellular enzymes are produced by roots and by microbes into the rhizosphere to break down organic molecules released through rhizodeposition (Wardle *et al*., 2004; Bardgett *et al*., 2008), providing available nutrients to the plant and maintaining rhizosphere functioning. Drought affects the diffusion of organic substrates in soil pores (Blagodatskaya *et al*., 2021; Zhang *et al*., 2023), which in turn alters the decomposition activity and is reflected in enzyme kinetics (Zhang *et al*., 2019). Parameters such as the maximum enzymatic rate (*V*_max_) and the affinity constant (*K*_m_) can reveal changes in the enzyme systems or the microbial community structure in response to drought (Acosta-Martínez *et al*., 2014; Fuchslueger *et al*., 2014). Part of the rhizosphere microbiome can thereby have beneficial effects on the plants, by influencing plant growth, abiotic stress tolerance, nutrient acquisition, pathogen resistance and even root system organization (Lugtenberg and Kamilova, 2009). Plant-beneficial bacteria include 1-aminocyclopropane-1-carboxylate (ACC) deaminase-carrying (*acdS*^+^) microorganisms, which are able to deaminate ACC, a precursor of the plant hormone ethylene (Fatma *et al*., 2022). The multiple ways in which *acdS*^+^-based microbial communities interact with plants are thereby influenced by environmental stressors and different genotypes (Ravanbakhsh *et al*., 2018). By preventing the formation of ethylene, the *acdS*^+^-based microbial community can support root elongation and with that nutrient acquisition and water uptake (Shahid *et al*., 2023). Glasshouse analysis of *acdS*^+^-based microbial community composition in maize rhizosphere showed that it is influenced strongly by soil properties and soil depth but only marginally by the presence of root hairs (Gebauer *et al*., 2021). Field-scale analyses showed that the composition of the *acdS*^+^-based microbial community differed according to field site, with the year of sampling having a low impact (Renoud *et al*., 2020), but the specific impact of abiotic stress in the field remains to be assessed.

In the field environment, plant–microbe interactions are not only influenced by different water levels over a specified time period, but over years, are influenced by soil and plants, and the history, or legacy, of the cultivated area (Williams and de Vries, 2020). For example, the impact of repeated maize planting on the rhizosphere microbiome is strong, leading to a decreased diversity of bacterial and fungal communities (Zhang *et al*., 2022) and an increase in maize disease incidence (Zhao *et al*., 2021). A possible explanation for this increased disease incidence during repeated maize planting is an increase in potential phytopathogenic fungi together with a decrease in protective microbes (Zhao *et al*., 2021), and this negative influence of repeated planting can be stronger in drought-experienced soil than in well-watered soil (Kaisermann *et al*., 2017; Lozano *et al*., 2022). Conversely, a drought history effect may be positive for maize, as it has been observed that maize inoculated with a microbiome derived from a water-limited agricultural site exhibits traits that enable it to withstand drought stress by conserving water (Carter *et al*., 2023). Furthermore, the functional consequences of naturally occurring soil microbiome variation are dependent on water availability, suggesting that future drying climates may dampen plants’ responsiveness to beneficial and/or pathogenic microbes.

There is still a lack of understanding of how different climatic conditions, such as droughts, affect maize roots and interactions with the rhizosphere microbiome, and for this reason we investigated the effect of annual weather variation on maize in a field experiment. It was set up in the frame of the research priority programme ‘Rhizosphere Spatiotemporal Organization – a Key to Rhizosphere Functions’, of which the growing seasons of three consecutive years, 2020, 2021 and 2022, were compared in this study. The years 2020 and 2022 can thereby be considered as dry years, compared to 2021, which was a year with above-average precipitation. To further disentangle the impact of root hairs and substrates, we included two maize genotypes, the B73 wild-type (WT) and the root hair-deficient mutant 3 (*rth3*), as well as the substrates loam (L) and sand (S). The studies included root gene expression, root enzyme activity and rhizosphere enzyme kinetic analyses for the total of 3 years and focused on maize plants during rapid vegetative growth at the BBCH19 growth phase, the nine-leaf stage. Based on the results of root gene expression and enzyme kinetics in the rhizosphere, analyses were carried out for the years 2021 and 2022 of the 16S rRNA- (bacteria and archaea), as well as *acdS*^+^-based (partial *acdS* gene) microbial community, all of which are strongly responsive to drought at the level of community composition (Gebauer *et al*., 2022). Investigations into fungi, other eukaryotes and viruses of the rhizosphere, potentially all interesting in light of plant drought response (Pratama *et al*., 2020; Lozano *et al*., 2021; Bazany *et al*., 2022) had to be put on hold due to budget restrictions. Although the *acdS* gene is present in some fungi, mainly within the phyla Ascomycota and Basidiomycota, its occurrence is much less frequent than in bacteria. It is only found in a few specific species of fungus, such as *Penicillium citrinum* and *Trichoderma asperellum*, which represent a small fraction of the total *acdS* gene pool (Singh *et al*., 2015). Therefore, we refer to both 16S rRNA- and *acdS*^+^-based microbial communities collectively as rhizosphere prokaryotes throughout the paper. Our study was guided by three working hypotheses. Expecting an adaptation of plant–soil legacy to previous dry years (Williams and de Vries, 2020; Carter *et al*., 2023), we hypothesized that (1) the drought response-related gene expression and rhizosphere enzyme activities during a dry year are less pronounced following a dry year than following a moist year. Genes related to drought and heat stress, mineral element uptake, cell wall restructuring, and defence and immunity are associated with this difference (Opitz *et al*., 2016; Li *et al*., 2017; Jiao *et al*., 2022; Kang *et al*., 2023). To counteract excessive ROS formation in the roots under drought (Kang *et al*., 2022), we furthermore hypothesized (2) higher root SOD activities in both dry years than the moist year, accompanied by highest activity levels after the moist year. Considering the effect of soil substrate on plant and rhizosphere microorganisms (Gebauer *et al*., 2021; Yim *et al*., 2022), and based on our previous work on maize roots from 2019 (Ganther *et al*., 2022) finding increased relative importance of substrate during low soil moisture, the third hypothesis states (3) that root gene expression and rhizosphere prokaryote composition are modulated by drought and substrate, and that the substrate effect is more pronounced in a dry year compared to a moist year.

## MATERIALS AND METHODS

### Experimental setup

The field soil plot experiment was conducted at a research station in Bad Lauchstädt (51°22′0″N, 11°49′60″E), central Germany, as part of the priority programme ‘Rhizosphere Spatiotemporal Organization – a Key to Rhizosphere Function’. A detailed description of the experimental setup is documented in Vetterlein *et al*. (2021). In this study, the second, third and fourth year (2020, 2021 and 2022) of a 5-year maize monoculture were compared, sampled during the vegetative growth period of maize at the nine-leaf stage BBCH19. Sowing took place on 28 and 29April 2020, 27 and 28 April 2021 and 26 and 27 April 2022 followed by 63–66 growing days until sampling, which was split into three days. In addition to the factor *year*, two *genotypes* differing in root hair elongation, *Zea mays* B73 WT and the *rth3* mutant, as well as the *substrates* loam (L) and sand (S), served as further experimental factors. Seeds of the *rth3* mutant (Wen and Schnable, 1994; Hochholdinger *et al*., 2008) and the corresponding WT were propagated at the experimental station Endenich of the Faculty of Agriculture of the University of Bonn. The loam substrate came from a haplic Phaeozem soil from Schladebach, Germany (51°18′31.41″N, 12°60′16.31″E). The sand substrate was obtained by mixing 16.7 % of the same loam with quartz sand from Quarzwerke Weferlingen, Germany. The soil plot experiment was performed in a two-factorial randomized block design with six biological replicates each, in individual plots. To compensate for substrate-related differences in nutrient availability, sand was fertilized with micronutrients and calcium (CaSO_4_.2H_2_O) and twice the amount of nitrogen (NH_4_NO_3_), phosphorus (CaHPO_4_), potassium (K_2_SO_4_) and magnesium (MgCl_2_.6H_2_O), compared to loam from 2019 until 2021. In 2022, loam received the same fertilization as sand. The fact that loam was not fertilized differently frpm sand in 2022 was due to higher plant nutrient uptake into plant biomass in loam than in sand (Vetterlein *et al*., 2021). Nutrient concentrations were determined in the youngest unfolded leaf at the four-leaf stage BBCH14, as described by Vetterlein *et al*. (2022).

### Root and rhizosphere sampling

The temperature was comparable at sampling times in 2020, 2021 and 2022, with mean daily temperatures of 21, 19 and 24 °C, respectively, but differing water content levels (Supplementary Data Figs S1 and S2). Sampling was randomized between treatments and restricted in time to limit variation caused by circadian changes in gene expression. For root and rhizosphere harvest, a 20 × 20 × 20-cm soil volume was excavated next to the maize plant. Plant roots were collected manually, with no differentiation between root type and part. Bigger soil clods (≥10 mm) attached to roots were removed. The soil obtained by gently brushing the collected roots was determined as the rhizosphere (Lucas *et al*., 2018), and stored at −20 °C until total community DNA extraction. The brushed off roots were washed by vortexing for 10 s in sterile 0.3 % (w/v) sodium chloride (NaCl) three times to remove remaining soil, followed by subsequent flash-frozing in liquid nitrogen. This resulted in six biological replicates of rhizosphere samples for each treatment. Four biological replicates of root samples were used for each treatment in 2020 and six biological replicates in 2021 and 2022. The *rth3* mutants grown in loam each had one biological replicate fewer, due to a difference in growth of one replicate. A reduction of replicates in 2020 was due to cost savings in RNA sequencing. The uneven replication could be handled by all statistical analyses described below.

### Root RNA sequencing

After pulverization in liquid nitrogen, RNA extraction was performed on 50 mg of root powder using the NucleoSpin RNA Plant Kit (Macherey-Nagel, Germany) according to the manufacturer's protocol. To obtain a larger total amount of RNA, it was eluted in the same 40 μL of RNase-free H_2_O twice. A NanoDrop 8000 spectrophotometer (Thermofisher Scientific, USA) and Bioanalyzer 2100 (Agilent, USA) were used to determine the RNA quality. All samples reached an RNA integrity number (RIN) of >8. The Novogene sequencing facility (Cambridge, UK) prepared 150-bp paired-end libraries and sequenced them on an Illumina NovaSeq 6000 sequencer (Illumina, USA). Trimmomatic v.0.39 (Bolger *et al*., 2014) with default settings was used to obtain clean reads, followed by mapping of sequences to the reference genome ‘*Zea mays* B73 RefGen_v5’ (MaizeGDB) using HISAT2 v.2.2.1 (Kim *et al*., 2015). To identify the number of mapped reads, the featureCounts software of Subread v.2.0.2 (Liao *et al*., 2014) was used. R v.4.3.0 (R-Core-Team, 2023) was used for statistical analyses. Differential gene expression analysis was performed using the package DESeq2 v.1.40.2 (Love *et al*., 2014). A Benjamini–Hochberg approach was used for *P*-value adjustment (Benjamini and Hochberg, 1995) and differentially expressed genes (DEGs) were only considered with *P-*adjust <0.05 and absolute log_2_ >1. To identify DEGs associated with specific functional categories (drought and heat stress, mineral element uptake, immunity and defense, exudation and secondary metabolism, and cell wall structure), we performed a keyword-based screening using the gene annotations of the reference genome. The full lists of DEGs with associated keywords are included in the Supplementary Data Tables S1–S5. Permutational analysis of variance (PERMANOVA) and variance partitioning analysis were performed using the ‘vegan’ (v.2.6–4) and ‘variancePartition’ (v.1.30.2) packages applying the ‘Adonis’ test with Euclidean distances (Hoffman and Schadt, 2016; Oksanen *et al*., 2025) using the VST-transformed counts of the DESeqDataSet object. RNAseq raw data were deposited in the National Center for Biotechnology Information (NCBI) Sequence Read Archive (SRA) under the BioProject PRJNA1195717.

### Superoxide dismutase activity

An enzyme activity assay was carried out in a slightly modified form according to Jiang and Zhang (2001). For protein extraction, 50 mg of root powder was added to 1 mL of extraction buffer, consisting of 50 mm sodium phosphate with 1 mm ethylenediaminetetraacetic acid (EDTA) and 1 % polyvinyl pyrrolidone (PVP) (pH 7.0). Extraction buffer containing the samples was then vortexed for 5 min and centrifuged at 4 °C for 20 min at 15 000 *g*. The supernatant was used as the enzyme extract. Superoxide dismutase activity was determined by measuring the inhibition of nitroblue tetrazolium (NBT) photoreduction induced by SOD. For this, 20 μL enzyme extract was transferred to a 96-well microtitre plate, together with solution I [140 μL potassium phosphate buffer (100 mm, pH 7) with EDTA (1 mm), 10 μL sodium carbonate (1.5 m), 20 μL L-methionine (0.2 m) and 10 μL riboflavin (60 μm)]. For the control, the enzyme extract was replaced by the extraction buffer. Finally, 50 μL of NBT (300 μm) was added and the reaction was immediately protected from light. The control measurement took place after a 10 min incubation under darkness, followed by measurement after 5 min of exposure to light. The corresponding absorbance was measured at 560 nm using a spectrophotometer (TECAN Infinite 200 pro, Tecan Life Sciences, Switzerland).

### Amplicon sequencing of 16S rRNA gene fragments

Only samples from the last two years 2021 (moist) and 2022 (dry) were used to analyse the rhizosphere prokaryote composition. Total community DNAs from the rhizosphere samples were extracted using the FastDNA Spin Kit and the GeneClean Spin Kit for soil (MP Biomedicals, Heidelberg, Germany) from ∼0.5 g soil. Sequencing libraries of the 16S rRNA gene fragments were created as previously described in Ganther *et al*. (2020) and Yim *et al*. (2022) using the primers Uni341F, 5′-CTAYGGGRBGCASCAG-3′ and Uni806R, 5′-GGACTACNNGGGTATCTAAT-3′ (Sundberg *et al*., 2013) to target the V3–V4 region of the 16S rRNA gene fragments. The amplicon libraries were created and sequencing was performed using a NovaSeq PE250 with 30 000 tags per sample at Novogene. The DADA2 pipeline (Callahan *et al*., 2016), and the SILVA SSU rel. 138.1 (Quast *et al*., 2013) databases were used to generate amplicon sequence variants (ASVs, 100 % identity) and the corresponding taxonomy data. The ASV abundance and taxonomy tables were imported using the phyloseq package (McMurdie and Holmes, 2013), and ASVs linked to chloroplasts, mitochondria and singletons were excluded for further analysis. The vegan package (Oksanen *et al*., 2025) was used to rarefy the number of sequences per sample to 12 825 (a sequence depth that covered all 16S diversity; data not presented) for alpha-diversity analysis. One replicate (WT grown in sand in 2021) was removed due to a considerably lower number of sequences. Beta-diversity was visualized using a non-metric multidimensional scaling (NMDS) based on the Bray–Curtis dissimilarity. PERMANOVA (the vegan package and 10 000 permutations) was used to compare the differences in beta-diversity between treatments. The Kruskal–Wallis test followed by Dunn's test (with Bonferroni *P*-value correction) were carried out to determine the significant effects of the treatments on alpha-diversity (Shannon index), and relative abundance of the 16S rRNA-based microbial community composition at the phylum level. The Wald test from Deseq2 was performed to test significant differences in relative abundance of genera affected by the year, based on log_2_ fold change with adjusted *P*-value <0.05 according to Benjamini–Hochberg (Benjamini and Hochberg, 1995). Only the 20 top most abundant genera (mean relative abundance ≥0.5 % across all treatments) whose relative abundance was significantly affected by the year (*P* < 0.01) were further tested if their relative abundance was also affected by the substrate and genotype using a Kruskal–Wallis test followed by Dunn's test (*P* < 0.05). All raw sequences of the 16S rRNA gene fragments were deposited in the NCBI with the project number PRJNA1195728.

### Amplicon sequencing of *acdS* gene fragments

Amplification was performed from the same DNA extract as before using the primers acdSF5 and acdSR8 (Bouffaud *et al*., 2018). The Quant-iT PicoGreen dsDNA Assay Kit (Invitrogen, Thermo Fisher Scientific, California, USA) was used to quantify the extracted DNA. Absorbance values were measured using a TECAN Infinite F200 PRO plate reader (TECAN, Switzerland). PCR amplification of *acdS* gene fragments was performed in an S1000 thermocycler (Bio-Rad Laboratories Inc., Hercules, CA, USA) using primers acdSF5 and acdSR8 (Bouffaud *et al*., 2018) and Kapa Hifi HotStart ReadyMix (KAPA-Biosystems, Wilmington, MA, USA). To avoid interference between PCR products, primer residues were removed according to the instructions for Illumina MiSeq sequencing. A NanoDrop was used to quantify the purified PCR products. Illumina MiSeq sequencing was then performed on a flow cell (Illumina Flow-Cell, Illumina Inc., CA, USA). Sequence analysis was performed with dadasnake v.0.10 (Weißbecker *et al*., 2020) using the DADA2 package (Callahan *et al*., 2016 ). A minimum base quality of 5 and a maximum expected error of 0.3 were used for filtering, with a minimum read length of 80 nucleotides for forward and reverse reads. Chimeric sequences were removed using the ‘consensus’ method of the package (Callahan *et al*., 2016). This resulted in a matrix of ASVs for each sample. After comparing the ASVs with an internal *acdS* database based on FunGene (Fish *et al*., 2013) and BLASTn (Bouffaud *et al*., 2018), unassigned ASVs were manually removed. The vegan package (v.2.6-4) (Oksanen *et al*., 2025) was used to rarefy the number of sequences per sample to 59 969 for alpha-diversity analysis. One replicate (WT grown in sand in 2021) was removed due to a considerably lower number of sequences. NMDS was used to visualize beta-diversity. PERMANOVA was performed using the ‘vegan’ package (v.2.6-4) to compare the differences in beta-diversity between treatments. The Kruskal–Wallis test followed by Dunn's test was used to determine significant differences between treatments on alpha-diversity and relative abundance at the phylum level. ASV genera were screened for differential abundance using DESeq2, based on log_2_fold changes with adjusted *P* < 0.05 (Benjamini and Hochberg, 1995). Raw sequences of the *acdS* gene fragments were deposited to the NCBI under the BioProject PRJNA1195717.

### Rhizosphere enzyme kinetics

The kinetics of enzymes related to organic carbon, nitrogen, phosphorus and microbial necromass turnover were measured in fresh rhizosphere samples by using fluorogenic substrates 4-methylumbelliferone-β-D-glucoside (CAS: 18997-57-4), 4-methylumbelliferone-phosphate (CAS: 22919-26-2), L-leucine 7-amino-4-methylcoumarin-hydrochloride (CAS: 62480-44-8) and 4-methylumbelliferone-*N*-acetyl-β-D-glucosaminide (CAS: 37067-30-4), purchased from Sigma Aldrich (Germany) to measure the activity of β-glucosidase (EC 3.2.1.21), acid phosphatase (EC 3.1.3.2), leucine aminopeptidase (EC 3.4.11.1) and *N*-acetylglucosaminidase (EC 3.2.1.14), respectively. To this end, 0.2 g of rhizosphere was suspended in 20 mL of MilliQ water using low-energy sonication (40 J s^−1^ output energy) for 1 min. Thereafter, enzyme reactions were measured in 96-well microplates using 50 μL of the rhizosphere suspension, 50 μL MES or Trizma of buffer [for 4-methylumbelliferone (MUF)-, or 7-amino-4-methylcoumarin-hydrochloride (AMC)-based substrates, respectively], and 100 μL of substrate dilution in a range of final concentrations: 0, 5, 20, 50, 75, 100, 200 and 400 μm (German *et al*., 2012). Fluorescence development was measured in dynamics at 360/465 nm excitation/emission wavelengths and a bandwidth of 35 nm with a plate reader (TECAN Infinite F200 Pro) during 150 min of incubation at room temperature, in the dark and under continuous orbital shaking. Standard curves for MUF and AMC products were obtained using the rhizosphere suspensions to counteract interactions between the product and the soil particles. Finally, we used the Michaelis–Menten (Michaelis and Menten, 1913) equation (Eqn. 1) to determine the maximum enzymatic rates (*V*_max_) and affinity constants (*K*_m_) of the analysed enzymes:


(1) $$\upsilon =\frac{V_{max}\;[S]}{K_{m}+[S]}$$

where ʋ is the rate of the enzymatic reaction (nmol MUF or AMC g^−1^ dry soil h^−1^), *V*_max_ is the maximum rate of the reaction (nmol MUF or AMC g^−1^ dry soil h^−1^), *S* is the substrate concentration (μm) and *K*_m_ is the affinity constant (µm), which corresponds to the substrate concentration at half of the maximum enzymatic rate. After applying the Michaelis–Menten equation, we used the rstatix package v.0.7.2 (Kassambara, 2023) in R to identify extreme outliers within *V*_max_ and *K*_m_: values above the third quartile plus three times the interquartile range, or below the first quartile minus three times the interquartile range. These could be associated with hotspots or coldspots of activity, overestimating or underestimating the mean activity in the rhizosphere, respectively. A detailed overview including raw and filtered values is provided in the Supplementary Data Table S6.

## RESULTS

To investigate the impact of varying precipitation levels on maize root gene expression and rhizosphere prokaryotes, the years 2020, 2021 and 2022 were selected, representing a dry, a moist and another dry year (Supplementary Data Fig. S3A). Cumulative precipitation at BBCH19, over our sample collection period, was lower in 2020 and 2022 than in 2021 (Fig. S3B). The year 2021 showed a higher volumetric water content in both substrates in the topsoil during the growth period and sampling (Figs S2 and S4) and a lower mean temperature compared to 2020 (Fig. S1). Differences in volumetric water content (VWC) between substrates are compensated for by their differing relationship between soil water content and soil water potential, resulting in differing field capacities of ∼23 % and 10 % VWC for loam and sand, respectively (Vetterlein *et al*., 2021). Taking this into account, plant-available water was relatively on a same level for both substrates and low in 2020 and 2022. The youngest unfolded leaf of the four-leaf stage was used for nutrient concentration analysis. Adjusting the fertilization for loam during 2022 (dry2) led largely to a maintenance of the nutrient ratio between the substrates. The remaining differences in plant nutrient concentrations between the years include an increase in calcium and phosphorus and decrease in potassium in 2022 (dry2) for both substrates. The concentration of plant nitrogen in loam was kept constant through adjusted fertilization. However, keeping the fertilization constant in sand resulted in a further decline in plant nitrogen status (Supplementary Data Table S7). For simplicity, the years 2020, 2021 and 2022 were designated as dry1, moist and dry2, respectively, and the two substrates and maize genotypes were termed as L_WT (loam, wild-type), L_*rth3* (loam, *rth3*), S_WT (sand, wild-type) and S_*rth3* (sand, *rth3*).

### Differential gene expression analysis of maize roots

**Fig. 1.**
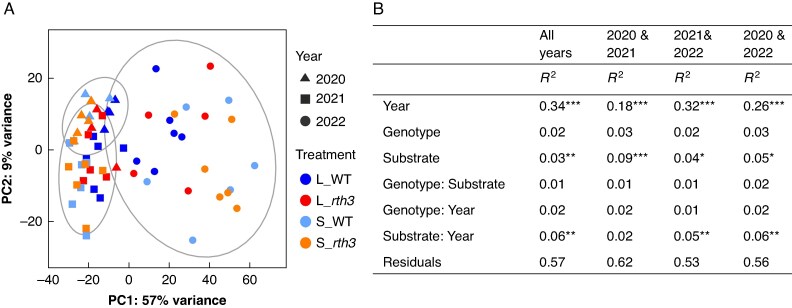
Principal component analysis (PCA) (A) and permutational analysis of variance (PERMANOVA) (B) of normalized gene expression levels in maize roots. B73 maize wild-type (WT) and root hair-deficient mutant 3 (*rth3*) grown on the substrates loam (L) and sand (S) were sampled in three years with different precipitation levels (2020 – dry, 2021 – moist, 2022 – dry). Ellipses are related to the factor ‘Year’. For 2020 n = 4, except for L_rth3 (n = 3). For 2021 and 2022 n = 6, except for L_rth3 (n = 5). The function ‘Adonis’ was used to display *R*^2^ and significance levels: *P < 0.05, **P < 0.01, ***P < 0.001. *R*^2^ values were rounded to two decimal places; total may not sum to 1 due to rounding.

Principal component analysis (PCA) of the full gene expression dataset revealed clustering by the factor year, with a higher separation between 2022 (dry2) and 2021 (moist) samples than between 2020 (dry1) and 2021 (moist) samples (Fig. 1A). This observation was supported by PERMANOVA results (Fig. 1B). For the full dataset, the factor year explained 34 % of the variance, but for the two-year comparisons, it explained 32 % of the variation between 2022 (dry2) and 2021 (moist), but 18 % of the variation between 2020 (dry1) and 2021 (moist) and 26 % between 2020 (dry1) and 2022 (dry2). Furthermore, the year 2022 (dry2) showed a high variation among the samples compared to 2020 (dry1) and 2021 (moist) (Fig. 1A). The factor substrate and the interaction of substrate and year also showed significant effects on the full gene expression dataset, with an explanatory value of 3 and 6 % respectively (Fig. 1B). Overall the effect of substrate was not very large, with the highest effects of 16, 13 and 15 % in 2020 (dry1), 2021 (moist) and 2022 (dry2), respectively (Table 1). The presence of root hairs did not significantly affect global gene expression. However, the gene with the mutation in *rth3*, *COBRA-like protein 7 (Roothairless 3)*, was consistently downregulated in the *rth3* mutant across all years and substrates (Supplementary Data Table S8). Closer analysis of the two-year comparisons revealed that the pattern of gene expression in 2022 (dry2) differed much more from that in 2021 (moist) than from that in 2020 (dry1). Specifically, between 2020 (dry1) and 2021 (moist), there were 1489 DEGs, of which 542 and 947 genes were up- and downregulated, respectively. In contrast, between 2022 (dry2) and 2021 (moist), we found more DEGs, 2990, of which 1246 were upregulated and 1744 were downregulated.

**Table 1.** Permutational analysis of variance (PERMANOVA) of the 16S rRNA-based and 1-aminocyclopropane-1-carboxylate deaminase (*acdS*^+^)-based microbial community structure of rhizosphere samples and root gene expression levels Samples were taken in two or three years with different precipitation (2020 – dry, 2021 – moist, 2022 – dry) from two maize genotypes, B73 wild-type and root hair-deficient mutant 3 *(rth3)* grown on the substrates loam and sand. The function ‘Adonis’ was used to display *R*^2^ and significance levels: **P < 0.01, ***P < 0.001.

	16S rRNA		*acdS* ^+^		Root gene expression		
	2021	2022	2021	2022	2020	2021	2022
	*R* ^2^	*R* ^2^	*R* ^2^	*R* ^2^	*R* ^2^	*R* ^2^	*R* ^2^
Substrate	0.27***	0.04	0.35***	0.27***	0.16**	0.13***	0.15**
Genotype	0.03	0.06	0.03	0.03	0.12	0.04	0.04
Residuals	0.67	0.86	0.59	0.67	0.69	0.80	0.78

**Fig. 2.**
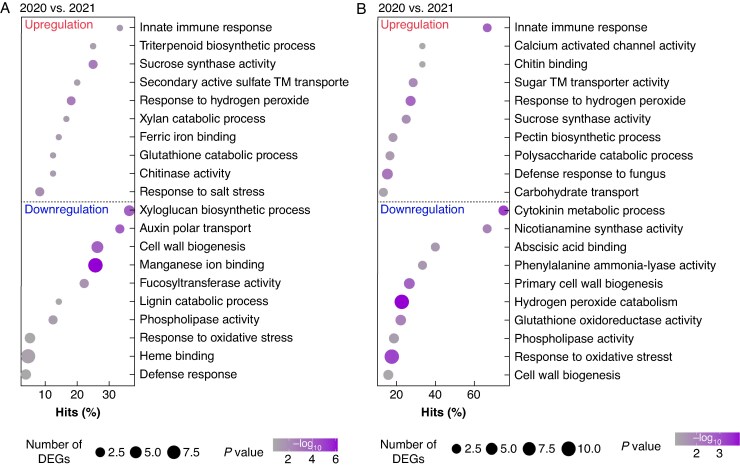
Gene ontology terms enriched in maize root up- and downregulated genes of B73 wild-type (WT) and root hair-deficient mutant 3 (*rth3)* grown on the substrates loam (L) and sand (S), between years with different precipitation levels. (A) Year 2020 (dry1) compared to 2021 (moist) and in (B) 2022 (dry2) compared to 2021 (moist). The upper half represents enriched GO terms for upregulated genes and the lower half for downregulated genes. Colour gradient represents −log_10_  *P-*value (*P* < 0.05) and the dot size represents the number of differentially expressed genes corresponding to each term. For 2020 n = 4, except for *rth3* grown in loam (n = 3). For 2021 and 2022 n = 6, except for *rth3* grown in loam (n = 5).

GO term enrichment analysis was performed to gain an overview of the functions of the DEGs. Overrepresented terms of the upregulated genes that appeared in both dry vs. moist comparisons were *innate immune response*, *sucrose synthase activity* and *response to hydrogen peroxide*, and a term related to *chitinase activity* (Fig. 2A, B). Enriched GO terms of the downregulated genes between both comparisons included *cell wall biogenesis*, *response to oxidative stress* and *phospholipase activity*. Specific analysis of 2020 (dry1) compared to 2021 (moist) detected overrepresented GO terms of the upregulated genes including secondary metabolite-related *triterpenoid biosynthetic process*, and nutrition-related *ferric ion binding* and *secondary active sulfate transmembrane transporter* (Fig. 2A). We furthermore found the term *response to salt stress* and ROS-related *glutathione catabolic process*, as well as cell wall-related *xylan catabolic process*. Conversely, among the overrepresented GO terms of the downregulated genes we found *manganese ion binding* and *auxin polar transport,* representing nutrition and hormone-related GO terms, respectively. Related to oxidative stress we found *heme binding*, accompanied by cell wall-related *lignin catabolic process* and *fucosyltransferase activity*. Specific analysis of 2022 (dry2) compared to 2021 (moist) detected overrepresented GO terms of the upregulated genes included the defence-related term *defense response to fungus*, five terms related to mineral element uptake and the cell wall-related term *pectin biosynthetic* process (Fig. 2B). Overrepresented GO terms of downregulated genes included *phenylalanine ammonia lyase-activity* related to secondary metabolism, and *nicotianamine synthase activity*, related to iron uptake. We furthermore found ROS-related *hydrogen peroxide catabolism* and *glutathione oxidoreductase activity*, as well as two terms related to cell wall biogenesis. *Cytokinin metabolic process* and *abscisic acid binding* represented hormone-related GO terms. Specific analysis of 2022 (dry2) compared to 2020 (dry1) revealed similarities to the comparison of 2022 and 2021 (dry2 vs. moist) (Supplementary Data Fig. S5; Fig. 2B). Overrepresented GO terms of upregulated genes included *defense response to fungus*, *sugar transmembrane transporter activity* and *pectin biosynthetic process*, whereas the overrepresented GO terms of downregulated genes included *phenylalanine ammonia-lyase activity*, *nicotianamine synthase activity*, *hydrogen peroxide catabolism* and *abscisic acid binding* (Fig. S5; Fig. 2B). Overrepresented terms among the upregulated genes that were present only between 2022 and 2020 (dry2 vs. dry1) included nutrition-related *potassium ion transport*, hormone-related *ethylene activated signaling*, as well as *mitochondrial calcium homeostasis* and *dioxygenase activity*, whereas among the downregulated genes the terms *phosphate ion transport*, *P-type divalent copper transport activity*, *acid phosphatase activity* and *cellular response to nitrogen starvation* as well as *cell wall macromolecule catabolism* occurred (Fig. S5).

**Fig. 3.**
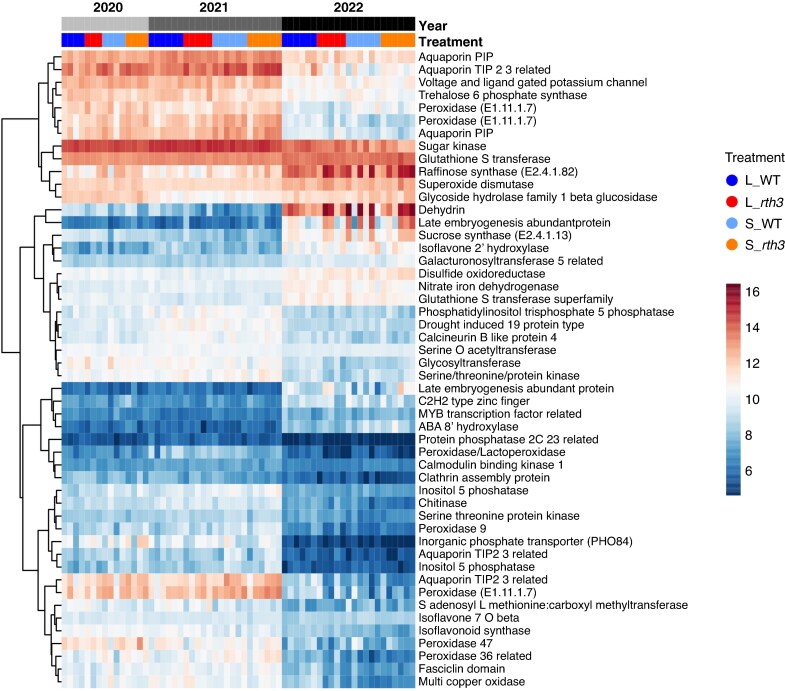
Cluster heatmap showing normalized expression levels of 50 out of 300 genes that explain the maximum variance in maize root gene expression. B73 maize wild-type (WT) and root hair-deficient mutant 3 (*rth3*) grown on the substrates loam (L) and sand (S) were sampled in three years with different precipitation levels (2020 – dry, 2021 – moist, 2022 – dry). For 2020 n = 4, except for *rth3* grown in loam (n = 3). For 2021 and 2022 n = 6, except for *rth3* grown in loam (n = 5).

A subsequent analysis of DEGs between dry years and the moist year focused on genes related to drought and heat stress (Supplementary Data Table S1), mineral element uptake (Table S2), immunity and defence (Table S3), exudation (Table S4) and cell wall structure (Table S5). Consistent with the total numbers of DEGs between the years, we found more individual DEGs related to drought and heat stress, mineral element uptake, exudation and cell wall in 2022 vs. 2021 (dry2 vs. moist) compared to 2020 vs. 2021 (dry1 vs. moist) (Tables S1–S5). Among the heat stress-related genes between 2020 and 2021 (dry1 vs. moist) we found *heat shock proteins*, *MYB like DNA binding proteins*, *NAC domain containing proteins* and *ethylene responsive transcription factors*, while between 2022 and 2021 (dry2 vs. moist), we also detected differentially expressed *aquaporins*, *dehydrins*, *late embryogenesis abundant proteins* and genes related to ABA (Table S1). DEGs between 2022 and 2020 (dry2 vs. dry1) included downregulated *aquaporin*s, but upregulated *heat shock proteins* and *late embryogenesis abundant proteins* in 2022 (dry2) (Table S1). Many mineral element uptake genes were downregulated between dry years and the moist year, such as *copper transport protein ATOX1* genes. In 2022 vs. 2020 (dry2 vs. dry1) we found downregulated *nicotianamine synthase* and *phosphate transporting ATPase* and *zinc/iron transporter* genes, as well as several genes related to phosphate and potassium transport (Table S2). DEGs related to plant immunity and defence included *thaumatins* and *pathogenesis related proteins*. Interestingly, the majority of these DEGs were downregulated between 2020 and 2021 (dry1 vs. moist), but upregulated between 2022 and 2021 (dry2 vs. moist) (Table S3). DEGs related to exudation (Table S4) between 2020 and 2021 (dry1 vs. moist) included downregulated *isoflavone 2 hydroxylase* and *methyltransferases,* but upregulated *MATE efflux family proteins*. In contrast, between 2022 and 2021 (dry2 vs. moist) *isoflavone 2 hydroxylases* were upregulated and the DEGs included flavonoid-, jasmonate-, neomenthol- and phenylalanine-related genes. From 2022 to 2020 (dry2 vs. dry1), a number of flavonoid and isoflavone biosynthesis-related genes were upregulated (Table S4). In addition, the cell wall formation-related DEGs included many downregulated *expansins* and *xyloglucan glucosyltransferases* between 2020 and 2021 (dry1 and moist) (Table S5). In contrast, the DEGs between 2022 and 2020 (dry2 vs. dry1) were few, with downregulated *expansins* and upregulated *xyloglucan xylosyltransferase*s (Table S5). The DEGs between the years dry1 (2020) and dry2 (2022) were further investigated by variance partitioning (Fig. 3). The transcript abundances of most of the genes explaining the variation between the dry years were lower in 2022 (dry2) than in 2020 (dry1) or 2021 (moist), and included *aquaporin*, *late embryogenesis abundant protein*, *phosphatase*, *isoflavonoid synthase* and *superoxide dismutase genes*. *Dehydrin*, *sucrose synthase* and *glutathione S transferase* were among the few genes with higher expression in 2022 (dry2) than in 2020 (dry1) or 2021 (moist) (Fig. 3).

To consider potential substrate effects between the years, PERMANOVA and GO term enrichment analysis were performed for loam and sand individually. In loam, PERMANOVA results revealed an effect of year of 24, 32 and 27 % between 2020 (dry1) and 2021 (moist), 2022 (dry2) and 2021 (moist), and 2022 (dry2) and 2020 (dry1), respectively (Supplementary Data Table S9). A similar result was observed in sand, with an effect of year of 21, 44 and 38 % between 2020 (dry1) and 2021 (moist), 2022 (dry2) and 2021 (moist), and 2022 (dry2) and 2020 (dry1), respectively (Table S9). In line with this, the number of DEGs was higher in 2022 (dry2) vs. 2021 (moist) than in 2020 (dry1) vs. 2021 (moist) in both substrates, with 3172 and 1495 in loam and 6477 and 1414 in sand, respectively. Overrepresented terms of the upregulated genes that appeared in both dry vs. moist comparisons and in both substrates were *response to heat*, *response to hydrogen peroxide* and a term related to chitin binding (Fig. S6). The term *defense response to fungus* was present among overrepresented terms of upregulated genes between 2022 (dry2) and 2021 (moist) in loam, and in both dry vs. moist comparisons in sand. Among overrepresented terms of downregulated genes, the term *response to oxidative stress* was present in both dry vs. moist comparisons in loam, and between 2022 (dry2) and 2021 (moist) in sand (Fig. S6). All comparisons contained terms related to cell wall (e.g. *cell wall biogenesis*) and mineral element uptake (e.g. *iron ion binding*) (Fig. S6). Specific analysis of 2022 (dry2) compared to 2020 (dry1) detected in both substrates among overrepresented terms of upregulated genes *sugar transmembrane transporter activity*, *carbohydrate transport* as well as a term related to ABA, whereas of downregulated genes were *cell wall biogenesis*, *iron ion binding*, *copper ion binding*, *response to oxidative stress* and *hydrogen peroxide catabolism* (Fig. S7).

### Root stress enzyme activity

**Fig. 4.**
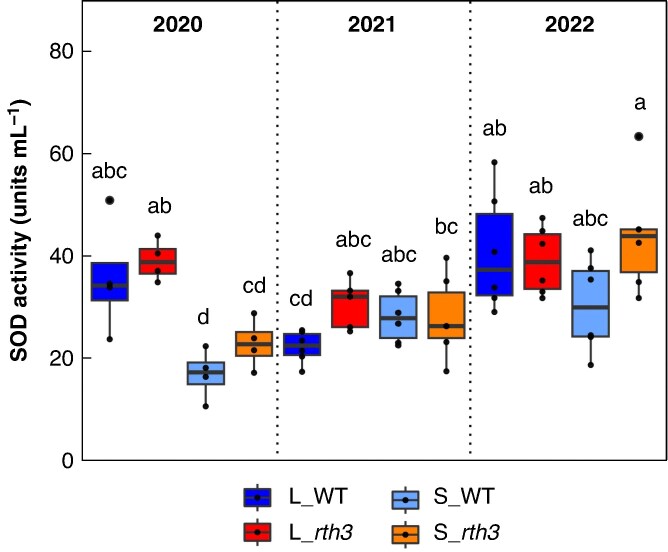
Superoxide dismutase (SOD) activity in maize roots. B73 maize wild-type (WT) and root hair-deficient mutant 3 (*rth3*) grown on the substrates loam (L) and sand (S) were sampled in three years with different precipitation levels (2020 – dry, 2021 – moist, 2022 – dry). Different letters indicate significant differences according to a linear mixed effects model and Tukey test (*P* < 0.05). For 2020 n = 4. For 2021 and 2022 n = 6, except for L_*rth3* (n = 5).

SOD activity analysis of maize roots revealed that only 2020 (dry1) showed a significant difference between the substrates, with higher SOD activity of L_WT compared to S_WT, as well as L_*rth3* compared to S_*rth3* (Fig. 4). S_WT is the only condition with a significant difference between 2020 (dry1) and 2021 (moist), showing a higher SOD activity in 2021 (moist). Significantly higher SOD activities in 2022 (dry2) compared to 2021 (moist) were observed for L_WT and S_*rth3.* The highest variation in SOD activity was observed in 2022 (dry2). Although not significant, a different trend of SOD activities was observed depending on the substrate. As roots grown in sand showed an increasing trend of SOD activities over the years, roots grown in loam showed higher SOD activities in both dry years (2020, 2022).

### Rhizosphere prokaryote composition

**Fig. 5.**
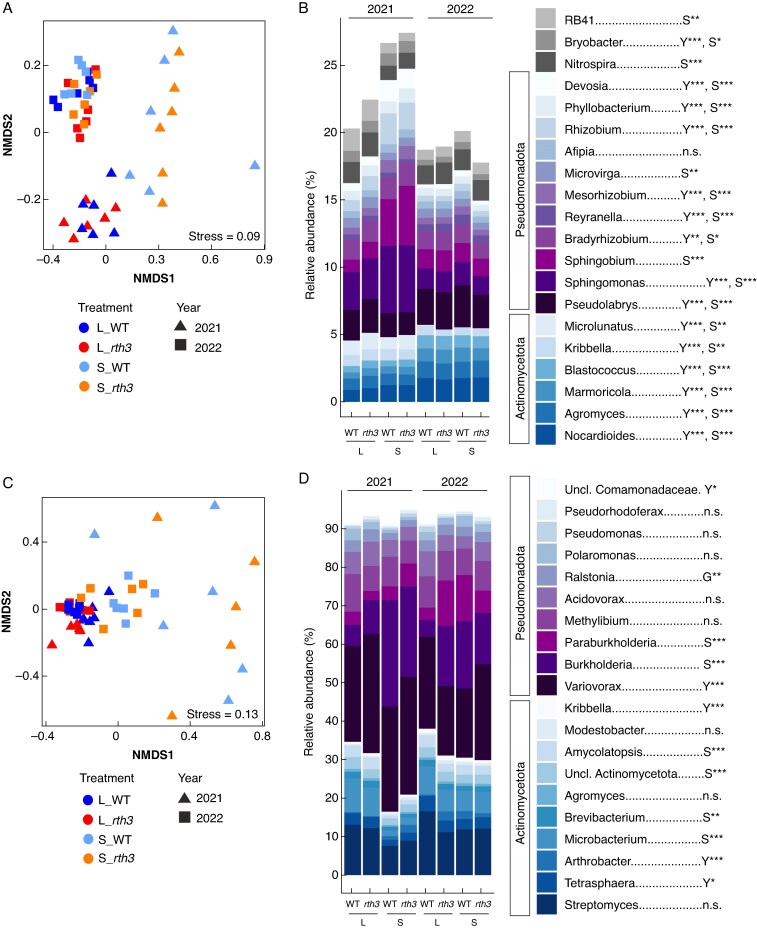
Non-metric multidimensional scaling (NMDS) (A, C) and relative abundance for the 20 most abundant genera of the 16S rRNA-based (A, B) and 1 aminocyclopropane-1-carboxylate deaminase (*acdS*^+^)-based (C, D) microbial community structure in the rhizosphere of maize between the years 2021 (moist) and 2022 (dry). B73 maize wild-type (WT) and root hair-deficient mutant 3 (*rth3*), grown on the substrates loam (L) and sand (S). Asterisks represent statistical significances of the factor year (Y), substrate (S) and genotype (G) with **P < 0.01, ***P < 0.001 and n.s. for not significant (Kruskal–Wallis test, followed by Dunn's test, n = 6, except for WT grown in sand, n = 5).

Having identified stronger differences in root gene expression levels between 2022 and 2021 (dry2 vs. moist) than between 2020 and 2021 (dry1 vs. moist), we decided to compare rhizosphere prokaryote structures between the two years 2022 (dry) and 2021 (moist), by examining the 16S rRNA-based and *acdS*^+^*-*based microbial community structure. 16S rRNA gene amplicon sequencing of prokaryotes identified that alpha-diversity (Shannon index) was significantly lower in the dry year (2022) compared to the moist year (2021) (Supplementary Data Fig. S8A). No effect of substrate or plant genotype on the Shannon index of the 16S rRNA-based microbial community was observed. NMDS showed significant clustering of beta-diversity for the moist (2021) and the dry year (2022) (Fig. 5A), as confirmed by the PERMANOVA test (*R*^2^ = 0.22) (Table 1). The effect of substrate on beta-diversity was also significant, but it was smaller than the effect of year (*R*^2^ = 0.09; Fig. 5A) and only significant in the moist year (Table 1). The relative abundance of Actinomycetota (former known as Actinobacteriota) and Chloroflexota was higher in dry than in moist years, but the relative abundance of Pseudomonadota (former known as Proteobacteria), Bacteroidota and Thermoproteota decreased (Fig. S9A). At a lower taxonomic rank, we found 15 of the 20 top most abundant bacterial genera (mean relative abundance ≥0.5 across all treatments) had a significant effect of year on their relative abundance (Fig. 5B). In line with the phylum level, the majority of bacterial genera affiliated to Actinomycetota (*Agromyces*, *Nocardioides*, *Marmoricola* and *Blastococcus*) were significantly increased in the dry year (2022) compared to the moist year (2021), whereas the genera belonging to Pseudomonadota (*Sphingomonas*, *Allorhizobium*, *Devosia*, *Phyllobacterium* and *Mesorhizobium*) decreased. The effect of substrate on the relative abundance of bacterial genera (e.g. *Sphingomonas*, *Sphingobium*, *Allorhizobium* and *Devosia*, all had higher relative abundance in sand than loam) were observed primarily in the moist year (2021) (Fig. 5B) as demonstrated by NMDS (above). The presence of root hairs had no effect on alpha- and beta-diversity or on the relative abundances of the 16S rRNA-based microbial community at taxonomic level (phylum and genus) (Table 2; Fig. 5B; Figs S8A and S9A).

**Table 2.** Permutational analysis of variance (PERMANOVA) of the 16S rRNA-based and 1-aminocyclopropane-1-carboxylate deaminase (*acdS*^+^)-based microbial community structure of rhizosphere samples. Samples were taken in two years with different precipitation (2021 – moist, 2022 – dry) from two maize genotypes, B73 wild-type and root hair-deficient mutant 3 (*rth3*), grown on the substrates loam and sand. The function ‘Adonis’ was used to display *R*^2^ and significance levels: **P < 0.01, ***P < 0.001.

	16S rRNA	*acdS* ^+^
	*R* ^2^	*R* ^2^
Year	0.22***	0.09***
Substrate	0.09***	0.24***
Genotype	0.02	0.02
Year:Substrate	0.07***	0.05**
Residuals	0.56	0.57

To target microorganisms carrying the *acdS* gene, which is involved in plant stress hormone ethylene regulation, amplicon sequencing of the *acdS* gene was used to assess whether the relative abundance of the *acdS*^+^*-*based microbial community is affected by drought. NMDS of *acdS* ASVs revealed a clear separation of samples between substrates, with high variation between samples in sand, particularly during the moist year 2021 (Fig. 5C). Conversely, a high similarity between samples was observed in loam, especially during the dry year (2022). Significant differences between substrates and years were found for alpha- (Supplementary Data Fig. S8B) and beta-diversity (Fig. 5D). The *acdS*^+^*-*based microbial community was dominated on a phylum level by Pseudomonadota, followed by Actinomycetota (Fig. S9B), with higher relative abundances of Actinomycetota in 2022 (dry) than in 2021 (moist) and for plant roots grown in loam than in sand. To better understand the changes in *acdS*^+^*-*based microbial community composition, the relative abundances of the 20 most abundant genera were compared between treatments and years (Fig. 5D). Members of *Burkholderia*, *Paraburkholderia* and *unclassified Actinomycetota* were less abundant in loam than in sand, and members of *Microbacterium*, *Brevibacterium*, *Amycolatopsis* and *Pseudorhodoferrax* were more abundant in loam than in sand. A higher relative abundance in the dry year (2022) than the moist year (2021) was found for *Tetrasphaera*, *Arthrobacter* and *Kribella*, but a lower abundance for *Variovorax* and *unclassified Comamonadaceae*. *Ralstonia* showed a significantly lower relative abundance in the rhizosphere of the *rth3* mutant than WT maize (Fig. 5D). These observations were supported by PERMANOVA, which identified substrate as the main driver of *acdS*^+^*-*based microbial community structure, followed by year, and the interaction of year and substrate (Table 2). Substrate explained 24 % of the variance, while the factor year and the interaction of substrate and year could only explain 9 and 5 % respectively. The effect of substrate remained high in both years when analysed separately, with a higher effect in 2021 (moist) than in 2022 (dry). The presence of root hairs did not show a significant effect according to PERMANOVA (Table 2).

### Rhizosphere enzyme kinetics

**Fig. 6.**
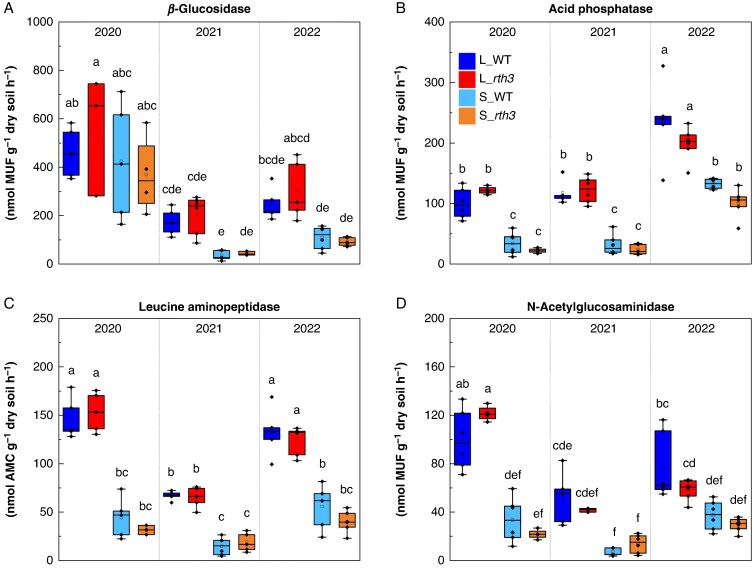
Maximum enzymatic rate (*V*_max_) of enzymes in the rhizosphere collected under B73 maize wild-type (WT) and root hair-deficient mutant 3 (*rth3*) grown on the substrates loam (L) and sand (S), in three years with different precipitation levels (2020 – dry, 2021 – moist, 2022 – dry). Different letters indicate significant differences according to a generalized linear model and Holm–Bonferroni method (*P* < 0.05), n = 3–6, as represented by data points. AMC, 7-amino-4-methylcoumarin; MUF, 4-methylumbelliferone.

The *V*_max_ of β-glucosidase decreased from 2020 to 2021 (dry1 vs. moist) in both substrates, and remained similar between 2021 and 2022 (moist and dry2) (Fig. 6A). The *V*_max_ of acid phosphatase increased from 2021 to 2022 (moist and dry2) for both soil substrates. The differences between substrates occurred each year as a lower *V*_max_ of acid phosphatase in sand compared to loam (Fig. 6B). Leucine aminopeptidase demonstrated a reduction in *V*_max_ from 2020 (dry1) to 2021 (moist) in loam, followed by an increase in *V*_max_ in 2022 (dry2) to similar values as in 2020 (dry1). In contrast, sand demonstrated stable *V*_max_ values of leucine aminopeptidase between years. The differences between substrates occurred each year as a lower *V*_max_ of leucine aminopeptidase in sand compared to loam (Fig. 6C). Finally, *V*_max_ of *N*-acetylglucosaminidase decreased from 2020 (dry1) to 2021 (moist) in loam, whereas sand substrate did not show differences between years. Differences between substrates occurred as a lower *V*_max_ of *N*-acetylglucosaminidase under both maize genotypes in 2020 (dry1) (Fig. 6D). Effects of plant genotype on differences in the *V*_max_ of the tested enzymes did not occur within the substrates in any year (Fig. 6). Regarding the affinity constant of enzymes (*K*_m_), a clear increase in affinity from 2020 (dry1) to the following years occurred for acid phosphatase. No clear trends over the years, or differences between substrates and genotypes were found for β-glucosidase, leucine aminopeptidase and *N*-acetylglucosaminidase enzyme affinities (Supplementary Data Fig. S10).

## DISCUSSION

### Drought responsive pattern in maize root gene expression

Enrichment analysis between dry and moist years (dry1 vs. moist, dry2 vs. moist) during the same growth stage revealed GO terms for immunity and defence, secondary metabolism, nutrition, oxidative stress and cell wall (Fig. 2), supported by groups of DEGs (Supplementary Data Tables S2–S5) and variance partitioning results (Fig. 3). In field-grown wheat, drought stress during the early reproductive period led to an enrichment of the GO term *oxidative stress*, in agreement with our result (Ma *et al*., 2017). In contrast to the enriched GO term for the upregulated genes of *triterpenoid biosynthesis* in maize, *flavonoid biosynthesis* was enriched in wheat. Interestingly, with maize we have previously suggested that upregulation of terpenoid biosynthesis-related gene expression also accompanies development from the nine-leaf stage BBCH19 to the tasseling stage BBCH59 (Ganther *et al*., 2022). In light of the current results, it is important to note that in the previous field analysis (2019) during BBCH59, drought symptoms occurred in maize leaves and the soil showed a low moisture level (Jorda *et al*., 2022), suggesting that drought stress may also have affected gene expression related to terpenoid biosynthesis in the previous study (Ganther *et al*., 2022). Maize gene expression patterns were in part consistent with short-term analyses of the drought response in maize. For example, as found here, Li *et al*. (2017) found several enriched GO terms related to defence and immunity in maize root hairs already following 5 d of drought, including the terms *immune response*, *defense response* and *chitinase activity*. In an experiment in the growth chamber, where WT maize and the *rth3* mutant were exposed to drought for 7 d, the term *defense response* was found to be enriched among downregulated genes (Hartwig *et al*., in press). The high occurrence of enriched GO terms and DEGs related to carbohydrates (Fig. 2) indicates an impact of drought on carbon allocation and processing (Guo *et al*., 2020; Hao *et al*., 2020; Opitz *et al*., 2014, 2016; Liu *et al*., 2015; Jiao *et al*., 2022; Kang *et al*., 2023; Hartwig et al., in press). Mineral nutrition may also be affected, as Jiao *et al*. (2022) and Opitz *et al*. (2016) found the GO term *potassium ion transmembrane transporter activity* and two differentially expressed potassium transport genes *kch1* and *kch4*, consistent with the observations here (Supplementary Data Table S2). The changes in cell wall-related gene expression levels are also consistent with previous results, indicating cell wall remodelling under drought. Under a water deficit of only 6 h, Opitz *et al*. (2016) found that the cell elongation-related gene *expa1* and the cell wall modification-related *xth1* were differentially expressed in the meristematic zone and the elongation zone of maize roots, respectively. Genes of these families were differentially expressed between both dry years and the moist year in this study (Table S5). Jiao *et al*. (2022) found after 7 h of water deficit the GO term *plant type cell wall*. Also, Kang *et al*. (2023) found 48 h after drought the enriched GO term *cell wall organization-* or *biosynthesis*, which fits with the term *cell wall biogenesis* represented between both dry years and the moist year (Fig. 2), supported by Hartwig *et al.* (in press), who found the GO term *cell wall* and Liu *et al*. (2015) who found two DEGs involved in cell wall-related biogenesis, after 1 week of drought. Note that variations in mean daily air temperatures during sampling from year to year (between 19 and 24 °C) may have contributed to variations in gene expression, which may be reflected, for example, in differences of the heat shock protein gene expression identified by RNA sequencing (Table S1). Under realistic field conditions, drought and heat stress often occur together, making it difficult to decipher their individual effects. Nevertheless, our results are partly consistent with previous observations of drought response not only under glasshouse experiments, but also in the field.

According to our first hypothesis, a greater effect of the factor year on gene expression levels with a higher number of DEGs occurred between 2022 and 2021 (dry2 and moist) than between 2020 and 2021 (dry1 and moist). Particular attention should be paid to the fact that 2020 (dry1) was the second dry year in a row, with cumulative precipitation levels below the average for this experimental site (Supplementary Data Fig. S3). A possible explanation for the different gene expression pattern in 2022 (dry2) could therefore be a stronger effect of drought after the moist period compared to the first dry year (2020) due to plant soil legacy (Williams and de Vries, 2020). In our study, fertilization was adjusted for loam during 2022 (dry2) to compensate for differences in plant nutrient uptake to plant biomass between loam and sand. Although the ratio of plant nutrient concentrations between loam and sand remained largely unchanged, significant differences of plant calcium, potassium and phosphate concentrations were observed from 2020 (dry1) to 2022 (dry2) for both substrates (Table S7), which means that a potential impact on the root transcriptome and rhizosphere prokaryotes cannot be ruled out. GO term enrichment patterns between 2022 (dry2) and 2020 (dry1) were examined for loam and sand separately (Fig. S7). For both substrates, the terms *copper ion binding* and *iron ion binding* were enriched among the downregulated genes. Neither of these terms indicates a limitation of nutrient supply during sampling, suggesting that the change in fertilization in loam served as an adjustment to the nutrient demand over the years (Fig. S7). A possible impact of repeated maize planting on the rhizosphere prokaryotes, and thus indirect the plants, should also be considered (Zhang *et al*., 2022; Giongo *et al*., 2024 ) and may be reflected in gene expression patterns of genes related to immunity and defence (Zhao *et al*., 2021). This is supported by an upregulation of genes related to immunity and defence in 2021 (moist) and 2022 (dry2) compared to the respective previous year (Table S3).

To ensure that the drought response was not specific to the loam or sand substrate, we separately examined the gene expression patterns between dry and moist years in the two soil substrates. According to the analysis including both substrates, a greater effect of the factor year on gene expression levels with a higher number of DEGs was observed between 2022 and 2021 (dry2 and moist) than between 2020 and 2021 (dry1 and moist) for loam and sand separately, too (Supplementary Data Table S9). Although the effect of year and the number of DEGs was higher in sand than in loam, both substrates showed a similar GO term enrichment pattern between both dry years and the moist year. This was reflected by the GO terms *response to heat* and *response to hydrogen peroxide* and accompanied by terms related to immunity and defence, cell wall restructuring, secondary metabolism and mineral element uptake (Fig. S6). These results are also highly consistent with the GO term pattern that includes both substrates (Fig. 2), suggesting that both substrates individually showed a drought response at the level of root gene expression.

### Substrate modulates maize root gene expression and superoxide dismutase activities

By looking at the three years as a whole, a different pattern of SOD activities for both substrates can be recognized (Fig. 4). SOD activities of roots grown in loam are higher in both dry years (2020, 2022), compared to the moist year (2021), which is in line with our second hypothesis, while SOD activities of roots grown in sand display an increasing trend over the years. The fact that this only applies to plant roots grown in sand could be due to a stronger influence of plants on the rhizosphere microbiome in sand, coming from a lower buffering capacity of sandy soils (Huang and Hartemink, 2020). Increased stress from repeated maize planting could also have an impact, as repeated planting can lead to lower maize yield, by changes not only in soil abiotic properties, but also the rhizosphere microbiome (Lal, 1997; Zhang *et al*., 2022). The year 2020 (dry1) was the only year with significantly higher SOD activities for roots grown in loam than sand (Fig. 4), which is accompanied by the highest substrate effect on root gene expression levels compared to 2021 (moist) and 2022 (dry2). Soil conditions, including water limitation and the soil texture differences between loam and sand, were shown to have a strong effect on water uptake in both WT and *rth3* maize (Cai *et al*., 2021). Together with an earlier onset of drought for maize plants grown in loam compared to sand, coming from larger plant sizes and thus higher water demand (Ganther *et al*., 2022; Jorda *et al*., 2022; Vetterlein *et al*., 2022), this would explain higher SOD activities for roots grown in loam. A possible drought adaptation of plant roots grown in sand is due to a higher root–shoot ratio (Gargallo-Garriga *et al*., 2014; Ganther *et al*., 2022) than in loam and could therefore also explain the differences in SOD activities between the two substrates. An impact of substrate on root gene expression levels was present in all comparisons, with the highest impact between 2020 and 2021 (dry1 vs. moist) (Fig. 1). Ganther *et al*. (2022) also observed a highly significant effect of substrate on root gene expression levels in maize and, as observed here, an increasing effect of substrate under drought. This is in line with the second part of our third hypothesis. Ganther *et al*. (2021, 2022) found a small impact of the presence of root hairs on root gene expression levels, and no significant impact was found in the present study. Additionally, no difference was detected for root stress enzyme activities. One reason for this could be the strong effect of the years and substrates, which may have masked the already small effect of root hair presence on gene expression. Overall, it should be noted that the samples were taken at only one time of the year and at only one maize growth stage, which on the one hand allows only a very selective comparison. On the other hand, by sampling at the same time of year and at the same stage of plant growth, we were able to study the weather and its different conditions as an isolated factor. Based on the observed soil water contents and the similar gene expression patterns between the two dry years, we believe that we have compared dry conditions represented by two years with moist conditions represented by one year.

### 16S-based and *acdS*^+^-based microbial community structures are influenced by the same drivers

Year had a main impact on rhizosphere prokaryotes, followed by substrate, confirming the first part of our third hypothesis, stating that rhizosphere prokaryotes are modulated by drought and substrate. The diversity and relative abundance of dominant taxa were affected by different water levels (drought) of 2021 and 2022 (moist and dry2) (Fig. 5A, B), consistent with previous studies (Hassani *et al*., 2018; Chun *et al*., 2022; Li *et al*., 2023). Drought may cause significant changes in carbon, nitrogen and O_2_ availability as well as pH for the 16S rRNA-based microbial community (Akinola *et al*., 2023; Jaeger *et al*., 2023). The decrease in the relative abundance of Gram-negative bacteria (i.e. *Sphingomonas* belonging to Pseudomonadota) and increase in the relative abundance of Gram-positive bacteria (e.g. *Nocardioides* and *Blastococcus* belonging to Actinomycetota) in the dry year (2022) compared to the moist year (2021) was consistent with previous studies, and these served as drought indicator taxa (Tiziani *et al*., 2022; Jaeger *et al*., 2023; Metze *et al*., 2023). The increased relative abundance of Gram-positive bacteria under drought could be explained by their thick peptidoglycan cell walls and sporulation ability (Xu *et al*., 2018).

In contrast to the 16S rRNA gene-based analysis, substrate, followed by year, had the greatest effects on the *acdS*^+^*-*based microbial community (Table 2). A large effect of substrate on maize rhizosphere *acdS*^+^*-*based microbial community diversity has also been demonstrated by Renoud *et al*. (2020) and Gebauer *et al*. (2021). How the *acdS*^+^*-*based microbial community responds to drought history was investigated in a glasshouse experiment by Gebauer *et al*. (2022), who showed that water deficit can induce a change in the *acdS*^+^*-*based microbial community that remains detectable during a subsequent period of low soil moisture in the following year. Looking more closely at the relative composition of the *acdS*^+^*-*based microbial community composition (Fig. 5D) and comparing that to other experiments focusing on the *acdS*^+^*-*based microbial community, a very similar phylogenetic distribution was found between the 20 most abundant genera (Bouffaud *et al*., 2018; Gebauer *et al*., 2021, 2022). Among them we found different relative abundances for genera of which members are known to be potentially plant growth-promoting rhizobacteria, for example *Streptomyces* (Schrey and Tarkka, 2008; Chukwuneme *et al*., 2020), *Burkholderia* (Naveed *et al*., 2014; Huang *et al*., 2017) and *Variovorax* (Chandra *et al*., 2019; Qi *et al*., 2022). In addition, a significant difference between WT and *rth3* mutant maize was found for the relative abundance of *Ralstonia*. Of this genus, *R. eutropha* stimulates maize nutrition and growth (Marques *et al*., 2010) (Fig. 5D). Small changes in the rhizosphere microbiome caused by the *rth3* mutation have been observed previously (Gebauer *et al*., 2022; Yim *et al*., 2022) and may be caused by an increased exudation rate per root surface (Santangeli *et al*., 2024).

Although year and substrate were the main drivers for maize root gene expression, 16S rRNA gene fragment and *acdS*^+^-based analysis, the effect of substrate on rhizosphere prokaryotes was higher during the moist year, which is contradictory to the root gene expression data (Tables 1 and 2) and conflicts with the second part of our third hypothesis, stating that the substrate effect is more pronounced in a dry year compared to a moist year. Additionally, both 16S rRNA-based and *acdS*^+^-based analysis showed the highest dissimilarities in ASV composition in sand during 2021 (moist) (Fig. 5A, C). Using the same substrates, Yim *et al*. (2022) found that substrate had a strong effect on the rhizosphere microbiome of both WT and *rth3* mutant maize plants grown in soil columns under well-watered conditions in a growth chamber, supporting our current findings. A smaller effect of substrate on the 16S rRNA-based microbial community in the dry year (2022) may be due a slower or reduced microbial growth rate. As reported by Metze *et al*. (2023), drought reduced the 16S rRNA-based microbial community growth rate by more than 90 %, allowing only about 4 % of the total community to grow. Additionally, drought has an impact on root exudation rate and exudate composition (Gargallo-Garriga *et al*., 2018; Canarini *et al*., 2019). Although drought tends to increase exudation rates (Zhang *et al*., 1995; Canarini *et al*., 2019), the response of rhizodeposition to drought can be very variable (Preece and Peñuelas, 2016). In this study, we found that the majority of DEGs related to exudation were downregulated in the dry year compared to the moist year (2022 vs. 2021) (Supplementary Data Table S4), indicating alterations in exudate composition, but providing no information on exudation rate. In fact, to achieve a coherent synthesis, future research should include root exudation analyses and microbial growth estimates.

In this study, our focus at the diversity level was on bacteria and archaea of the rhizosphere. The response of bacterial, fungal and protist communities in the plant rhizosphere to water deficiency stress depends on the microbial group. Generally, drought has a stronger impact on prokaryotes than on fungi or protists in terms of rhizosphere structural diversity and community composition (Dai *et al*., 2019; Bazany *et al*., 2022). However, we agree that responses by different kingdoms are important for the response to drought, and found support for this in our root gene expression data. For example, the repeatedly occurring GO term *defense response to fungus* also indicates potential shifts in the rhizosphere fungal community (Fig. 2B; Supplementary Data Figs S5 and S6B–D). Changes in fungal community composition have been associated with the response of the rhizosphere to drought (Lozano *et al*., 2021). Drought-resistant wheat, for example, has a more diverse range of fungal taxa than drought-sensitive wheat, and the networks of microbial interactions between bacteria and fungi in the drought-resistant variety are more complex than in the sensitive variety (Yue *et al*., 2024). The relative importance of environmental stress and host factors can vary between microbial kingdoms. Water deficit treatment had a stronger influence on bacterial composition than host species, whereas the opposite was true for protists. Water deficit also impacted intra- and inter-kingdom microbial associations, with protist taxa forming a distinct cluster under deficit conditions (Bazany *et al*., 2022). The composition and diversity of viruses in the rhizosphere remain largely unexplored, but it is known that rhizosphere phages exert a control over rhizosphere bacteria (Pratama *et al*., 2020). Liu *et al*. (2025) demonstrated that drought intensifies negative prokaryote–phage interactions, resulting in an increased number of negative links in the prokaryote–phage co-occurrence network and a greater frequency and diversity of anti-phage defence systems in prokaryotic genomes. This led to greater top-down control of typical soil K-strategists, including Acidobacteriota and Chloroflexota.

### Enzyme kinetics responded to drought and soil substrate

The effect of drought on enzyme activities was enzyme-specific, most evident in acid phosphatase and leucine aminopeptidase. The observed increase in the *V*_max_ of acid phosphatase in loam after the moist year (2021) (Fig. 6B and C) could be explained by the increased affinity of enzymes (Supplementary Data Fig. S10). Changes in phosphatase activity are strongly associated with climatic conditions as well as soil type (Margalef *et al*., 2017). Drought can decrease phosphatase activity in soil (Sardans *et al*., 2008; Zhang *et al*., 2020), due to the energy and nitrogen requirements for the production of this class of extracellular enzymes (Houlton *et al*., 2008). However, recurrent drought events may engender a resilience of enzyme activities to drought due to an altered microbiome structure and functions (Canarini *et al*., 2021). This could be reflected in our study as an increased affinity of acid phosphatase in 2021 (moist), following 2 years of drought in the same field experiment. This increased affinity, indicating altered enzyme systems, may have resulted in the increased *V*_max_ of acid phosphatase in 2022 (dry2) as compared to the previous dry year (2020). In contrast, leucine aminopeptidase exhibited a reduction in *V*_max_ during the moist year (2021), which may support the hypothesis that microbial communities have adapted to drought conditions. This correlated with the changes in rhizosphere prokaryote composition, with a greater abundance of Gram-positive than Gram-negative bacteria. The quality of sand substrate results in certain limitations, including a larger pore size, a lower retention capacity for water and nutrients, and a lower total organic carbon and total nitrogen content (Huang and Hartemink, 2020; Vetterlein *et al*., 2021). These characteristics explain the lower *V*_max_ of leucine aminopeptidase and acid phosphatase over time in sand compared to loam, as well as the negligible response of enzymes in this substrate to climatic changes (only an increase in *V*_max_ of acid phosphatase was observed in 2022 compared to the previous 2 years).

## CONCLUSIONS

Our gene expression analyses, combined with analyses of potential rhizosphere enzyme activity and rhizosphere prokaryote diversity, illustrate how maize responds to prolonged drought and how this is translated into the rhizosphere. We found that the patterns between the two dry years and the moist year were distinct and specific to each dataset. Although both dry years revealed drought-related patterns, a stronger response was shown after the moist year, while root stress levels showed different results between the substrates, leading to associated changes in the rhizosphere prokaryotes. This highlights the complex role of soil and precipitation history effects in shaping plant–microbe interactions. Although our experimental design allowed us to elucidate the influence of different weather conditions, it should be noted that one sampling time point per year provides only very selective insight. For example, the role of rhizodeposition and temperature variations should be investigated in the future. The strong impact of substrate on root gene expression and the 16S rRNA-based as well as the *acdS*^+^*-*based microbial community highlighted interactive effects between the maize roots and the rhizosphere prokaryotes. In support of our earlier observations from a single year, root hairs had little influence on these dynamics, suggesting that other mechanisms play a more dominant role. Future work will focus on the effects of agricultural practices, such as repeated planting of maize, on these interactions.

## Supplementary Material

mcaf180_Supplementary_Data

## SUPPLEMENTARY MATERIALS

Figure S1. Temperature at the weather station in Bad Lauchstädt, Germany, and soil volumetric water content on sampling days. Figure S2. Volumetric water content on sampling days for each treatment. Figure S3. Precipitation at the weather station in Bad Lauchstädt, Germany, in the years 2018–2023 and at growth stage BBCH14 and BBCH19 in the years 2020, 2021 and 2022. Figure S4. Volumetric water content from sowing until harvest. Figure S5. Gene ontology terms enriched in maize root up- and downregulated genes between 2022 and 2020. Figure S6. Gene ontology terms enriched in maize root up- and down-regulated genes between the two dry years and a moist year for the two substrates separately. Figure S7. Gene ontology terms enriched in maize root up- and down-regulated genes between the two dry years for the two substrates separately. Figure S8. Shannon index of rhizosphere prokaryote structure. Figure S9. Relative abundance of rhizosphere prokaryote structure. Figure S10. Affinity constant (*K*_m_) of rhizosphere enzymes. Table S1. Differentially expressed maize root genes related to drought and heat stress. Table S2. Differentially expressed maize root genes related to mineral element uptake. Table S3. Differentially expressed maize root genes related to immunity and defence. Table S4. Differentially expressed maize root genes related to exudation and secondary metabolism. Table S5. Differentially expressed maize root genes related to cell wall structure. Table S6. Raw and filtered values of maximum enzymatic rate (*V*_max_) and affinity constant (*K*_m_) of rhizosphere enzymes. Table S7. Nutrient concentrations in the youngest unfolded leaf of maize plants. Table S8. Genes associated with the *rth3* (root hair defective 3) mutation between B73 maize wild-type (WT) and *rth3* mutant roots. Table S9. Permutational analysis of variance (PERMANOVA) of root gene expression levels for the two substrates separately.
