# Supplementary material for: Responses of maize roots, rhizosphere enzyme kinetics and prokaryote diversity to alternating precipitation: insights from a three-year field study
Source: Ann Bot. 2025 Aug 6;136(5-6):1081–99. doi: 10.1093/aob/mcaf180 (PMC12682858; doi:10.1093/aob/mcaf180)
Supplement: mcaf180_Supplementary_Data [file mcaf180_supplementary_data.zip › Supplementary_Figures_S1-S10.pdf]

Responses of Maize Roots, Rhizosphere Enzyme Kinetics, and Prokaryote Diversity to Alternating Precipitation: Insights from a Three-Year Field Study

SUPPLEMENTARY DATA – FIGURES

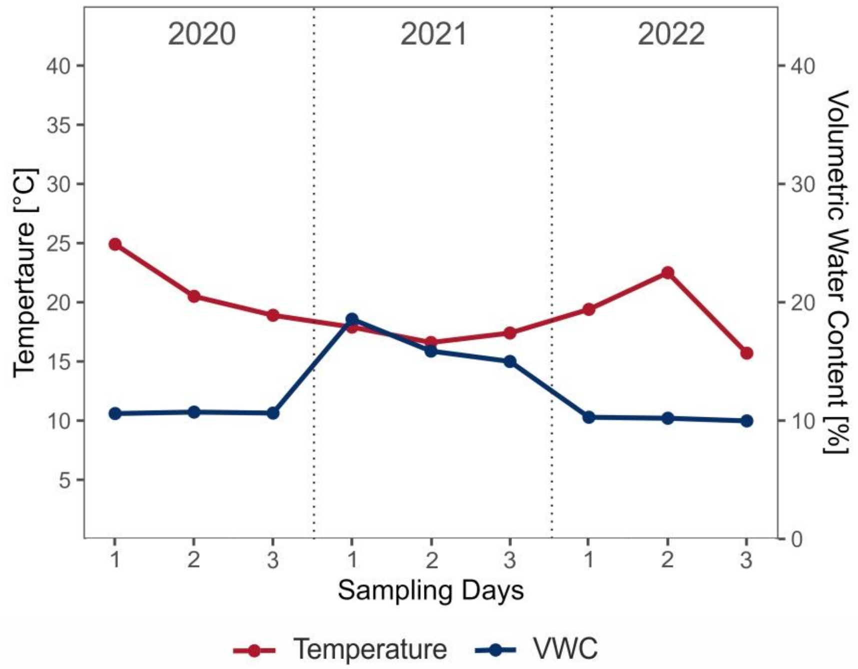

FIG. S1. Air temperature (red) and soil volumetric water content (blue) at 9am on sampling days in Bad Lauchstädt, Germany in 2020, 2021 and 2022. The temperature data was obtained from the DWD Climate Data Center (CDC) and the volumetric water content was measured as described by Jorda et al. (2022) at a depth of 10cm.

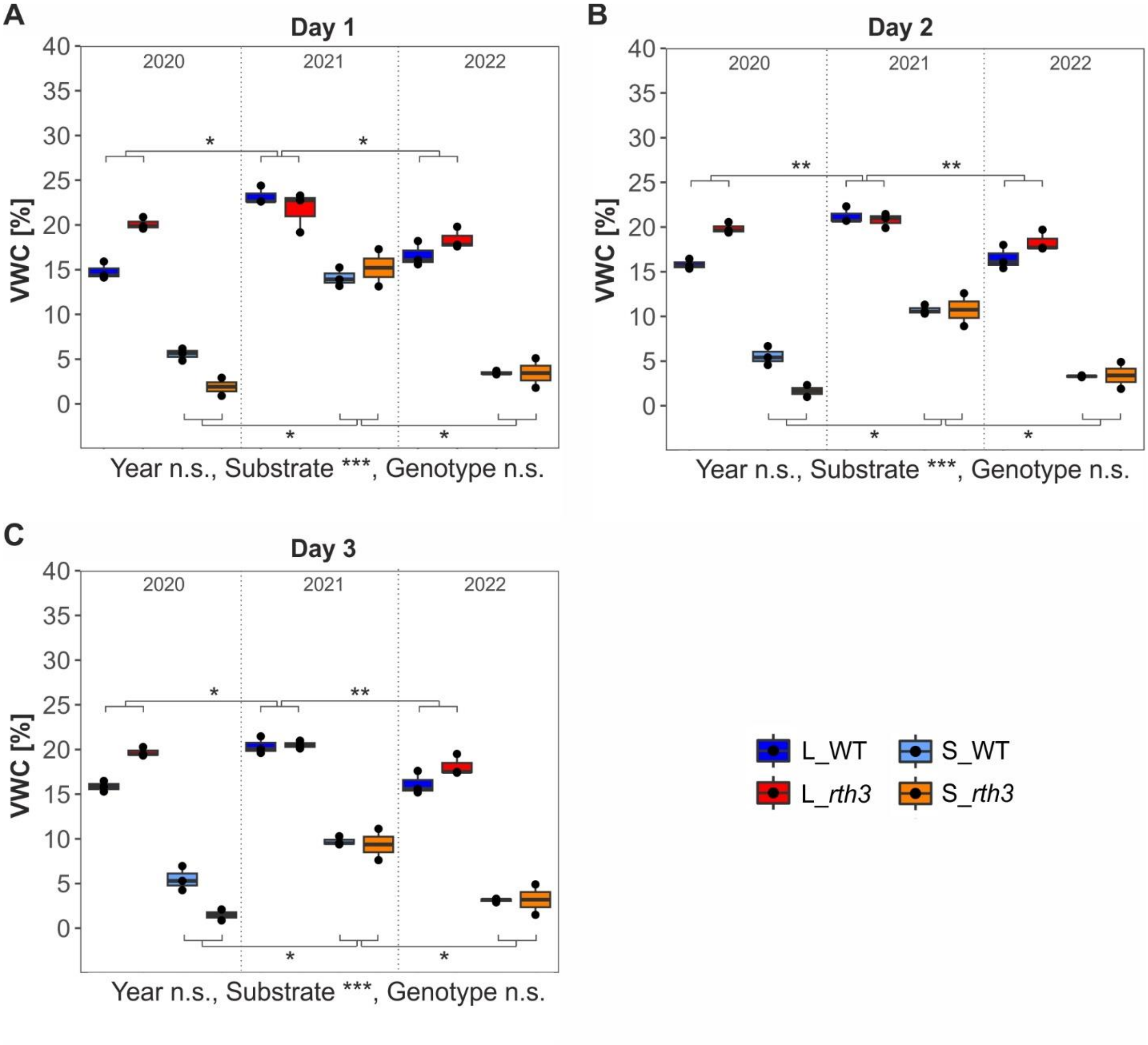

FIG. S2. Volumetric water content (VWC) on sampling day 1 (A), 2 (B) and 3 (C) of B73 maize wild type (WT) and the root hair deficient mutant 3 (*rth3*) plants, grown on the substrates loam (L) and sand (S) of three years with different precipitation levels (2020 - dry, 2021 - moist, 2022 - dry). Water content was measured as described by Jorda et al. (2022) at 9 o'clock, at a depth of 10 cm. Asterisks indicate significances between the factor year, substrate and genotype, for n = 3 technical replicates (Kruskal-Wallis Test, followed by Wilcoxon rank-sum Test; $p < 0.05$). *, *** and n.s.; $p < 0.05$, < 0.001 and non-significant, respectively.

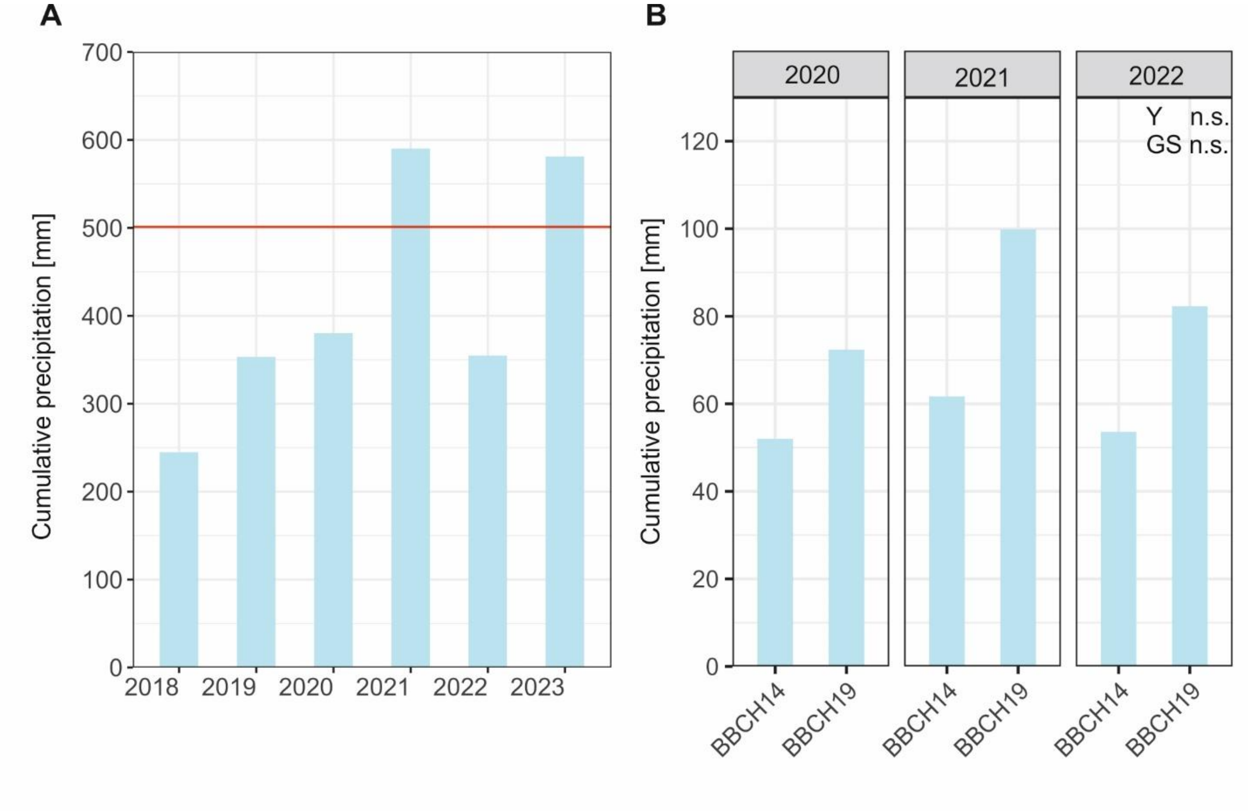

FIG. S3. Cumulative precipitation at the weather station in Bad Lauchstädt, Germany, in the years 2018 to 2023 (A) and at growth stage BBCH14 and BBCH19 in 2020, 2021 and 2022 (including irrigation) (B). Red line indicates the average cumulative precipitation of the last 30 years. Data was obtained by the DWD Climate Data Center (CDC). For (A) n = 365, except for 2020 with n = 366. Because there were missing values for August and September in 2018, the average values from the closest two weather stations (Querfurt-Mühle, ~ 23km west and Leipzig/Halle ~ 23km east) were determined here. n.s.; no significance (p < 0.05) according to Kruskal-Wallis Test (performed for both growth stages separately); Y, year; G, growth stage, n = 43 for BBCH14 and n = 21 for BBCH19 (B).

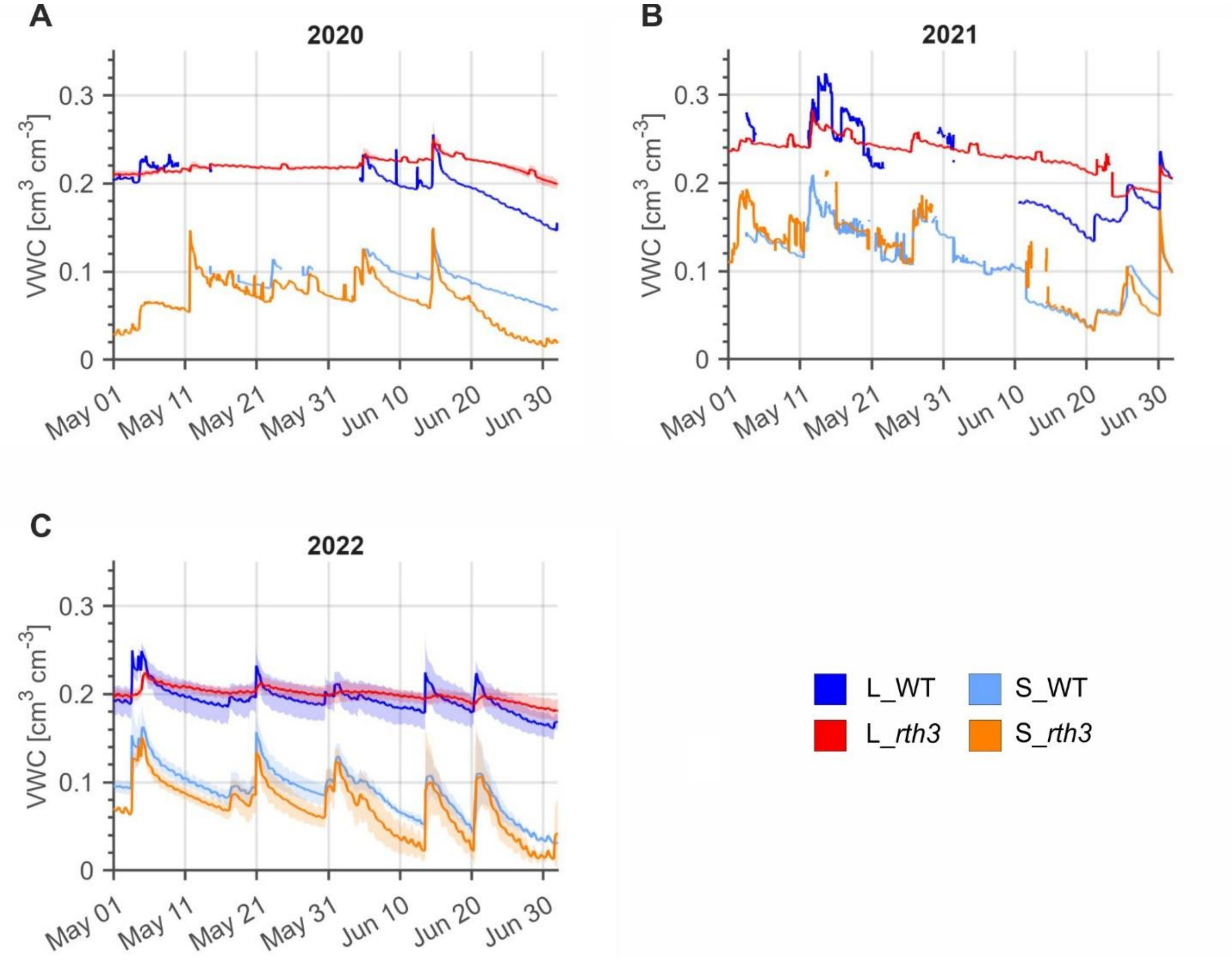

FIG. S4. Volumetric water content (VWC) at a depth of 10 cm in experimental plots measured from sowing until harvest at BBCH19 in three years with different precipitation levels (2020 - dry, 2021 - moist, 2022 - dry). Plots contained B73 maize wild type (WT) and the root hair deficient mutant 3 (*rth3*) plants, grown on the substrates loam (L) and sand (S). Water content was measured as described by Jorda et al. (2022).

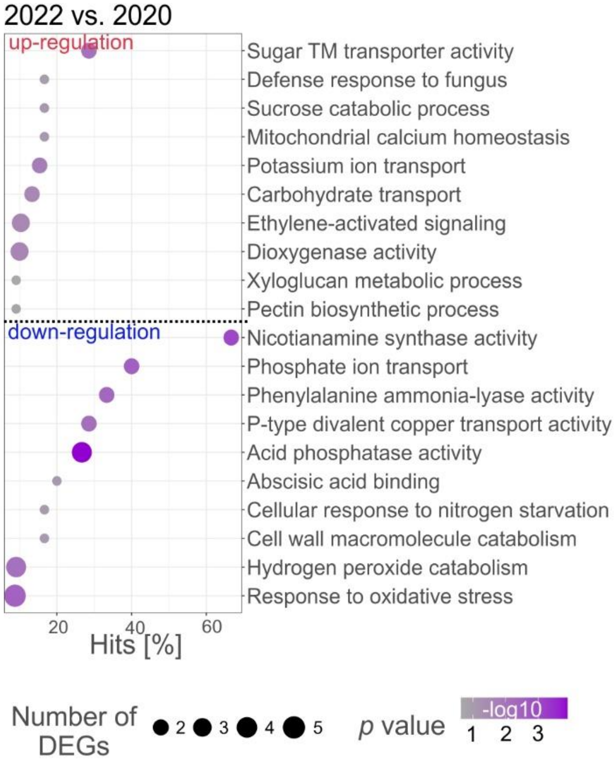

Fig. S5. Gene ontology terms enriched in maize root up- and down-regulated genes of B73 wild type (WT) and the root hair deficient mutant 3 (*rth3*) plants grown on loam and sand, between years with similar low precipitation levels. The upper half represents enriched GO terms for upregulated genes and the lower half for downregulated genes. Color gradient represents -log10 *p* value ($p < 0.05$) and the dot size represents the number of differential expressed genes corresponding to each term. In 2020 n = 3 for plants grown on loam and n = 4 for plants grown in sand; in 2022 n = 6, except for *rth3* mutant grown in loam (n = 5).

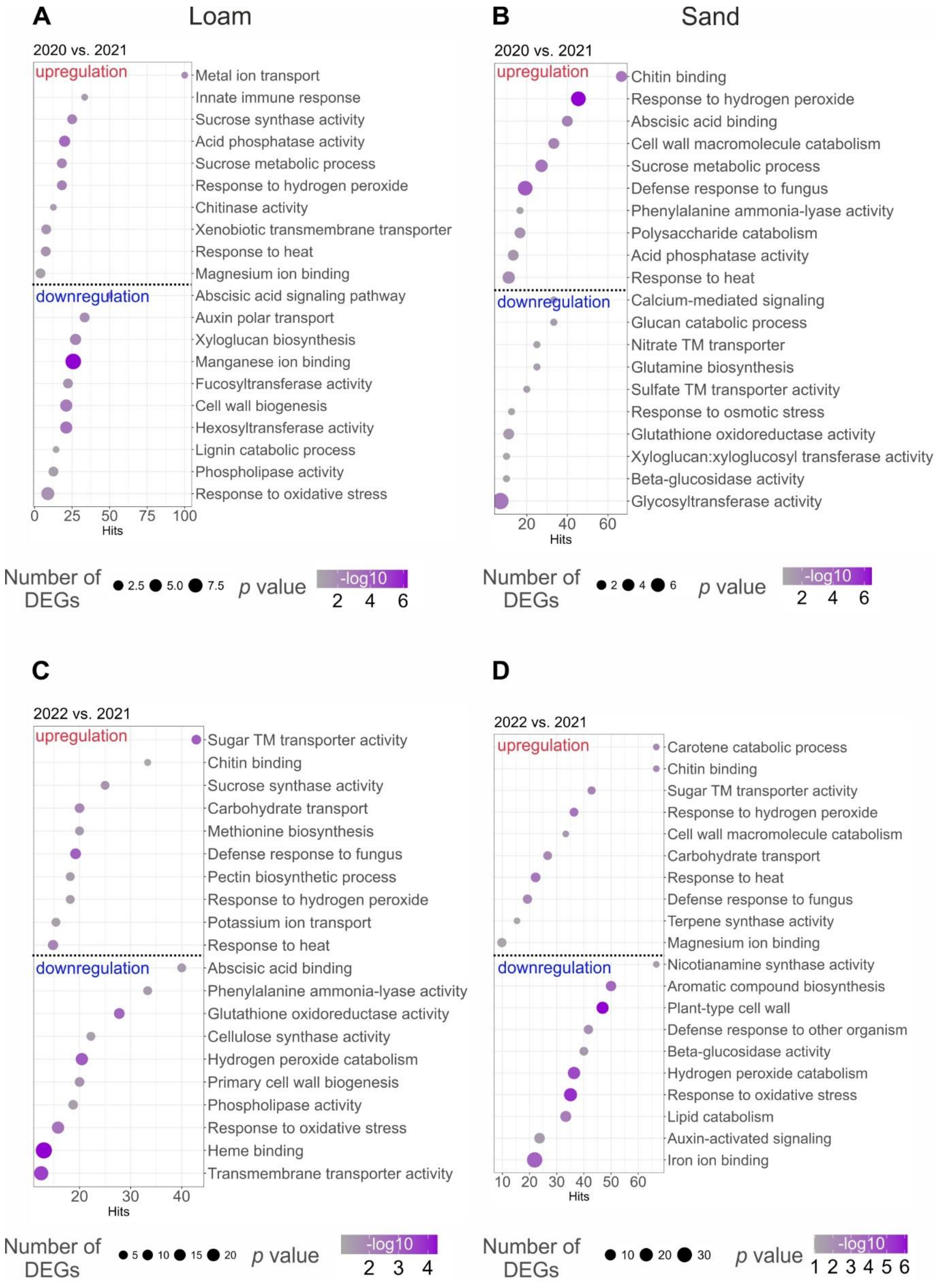

A
Loam
2020 vs. 2021
upregulation
Metal ion transport
Innate immune response
Sucrose synthase activity
Acid phosphatase activity
Sucrose metabolic process
Response to hydrogen peroxide
Chitinase activity
Xenobiotic transmembrane transporter
Response to heat
Magnesium ion binding
downregulation
Abscisic acid signaling pathway
Auxin polar transport
Xyloglucan biosynthesis
Manganese ion binding
Fucosyltransferase activity
Cell wall biogenesis
Hexosyltransferase activity
Lignin catabolic process
Phospholipase activity
Response to oxidative stress
0 25 50 75 100
Hits
Number of DEGs
2.5 5.0 7.5
p value
-log10
2 4 6
B
Sand
2020 vs. 2021
upregulation
Chitin binding
Response to hydrogen peroxide
Abscisic acid binding
Cell wall macromolecule catabolism
Sucrose metabolic process
Defense response to fungus
Phenylalanine ammonia-lyase activity
Polysaccharide catabolism
Acid phosphatase activity
Response to heat
downregulation
Calcium-mediated signaling
Glucan catabolic process
Nitrate TM transporter
Glutamine biosynthesis
Sulfate TM transporter activity
Response to osmotic stress
Glutathione oxidoreductase activity
Xyloglucan:xyloglucosyl transferase activity
Beta-glucosidase activity
Glycosyltransferase activity
20 40 60
Hits
Number of DEGs
2 4 6
p value
-log10
2 4 6
C
2022 vs. 2021
upregulation
Sugar TM transporter activity
Chitin binding
Sucrose synthase activity
Carbohydrate transport
Methionine biosynthesis
Defense response to fungus
Pectin biosynthetic process
Response to hydrogen peroxide
Potassium ion transport
Response to heat
downregulation
Abscisic acid binding
Phenylalanine ammonia-lyase activity
Glutathione oxidoreductase activity
Cellulose synthase activity
Hydrogen peroxide catabolism
Primary cell wall biogenesis
Phospholipase activity
Response to oxidative stress
Heme binding
Transmembrane transporter activity
20 30 40
Hits
Number of DEGs
5 10 15 20
p value
-log10
2 3 4
D
2022 vs. 2021
upregulation
Carotene catabolic process
Chitin binding
Sugar TM transporter activity
Response to hydrogen peroxide
Cell wall macromolecule catabolism
Carbohydrate transport
Response to heat
Defense response to fungus
Terpene synthase activity
Magnesium ion binding
downregulation
Nicotianamine synthase activity
Aromatic compound biosynthesis
Plant-type cell wall
Defense response to other organism
Beta-glucosidase activity
Hydrogen peroxide catabolism
Response to oxidative stress
Lipid catabolism
Auxin-activated signaling
Iron ion binding
10 20 30 40 50 60
Hits
Number of DEGs
10 20 30
p value
-log10
1 2 3 4 5 6

FIG. S6. Gene ontology terms enriched in maize root up- and down-regulated genes of B73 wild type (WT) and the root hair deficient mutant 3 (*rth3*) plants grown on loam (A, C) and sand (B, D), between years with different precipitation (2020 – dry, 2021 – moist, 2022 – dry). The upper half represents enriched GO terms for upregulated genes and the lower half for downregulated genes. Color gradient represents -log10 p value ($p < 0.05$) and the dot size represents the number of differential expressed genes corresponding to each term. In 2020 n = 3 for plants grown on loam and n = 4 for plants grown in sand; in 2022 n = 6, except for *rth3* mutant grown in loam (n = 5).

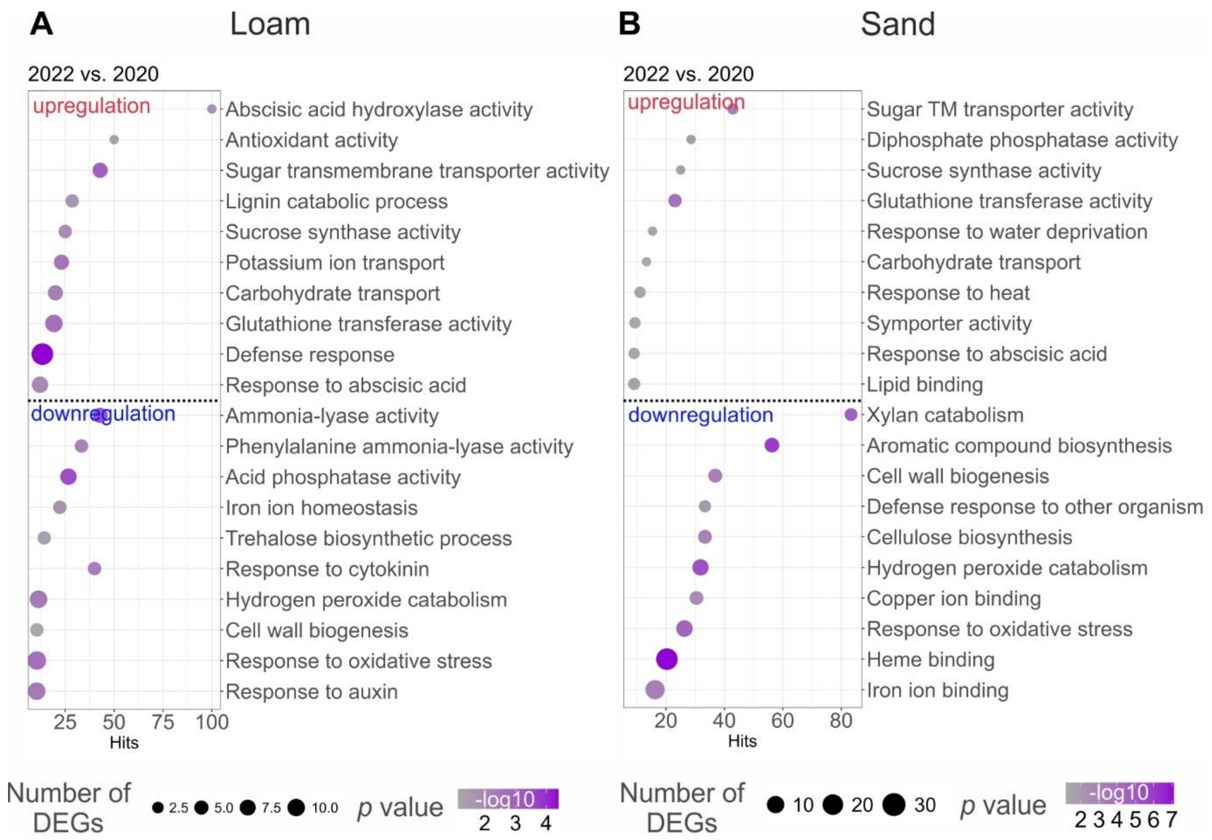

FIG. S7. Gene ontology terms enriched in maize root up- and down-regulated genes of B73 wild type (WT) and the root hair deficient mutant 3 (*rth3*) plants grown on loam (A, B) and sand (B, D), between years with similar low precipitation levels. The upper half represents enriched GO terms for upregulated genes and the lower half for downregulated genes. Color gradient represents -log10 *p* value ($p < 0.05$) and the dot size represents the number of differential expressed genes corresponding to each term. In 2020 n = 3 for plants grown on loam and n = 4 for plants grown in sand; in 2022 n = 6, except for *rth3* mutant grown in loam (n = 5).

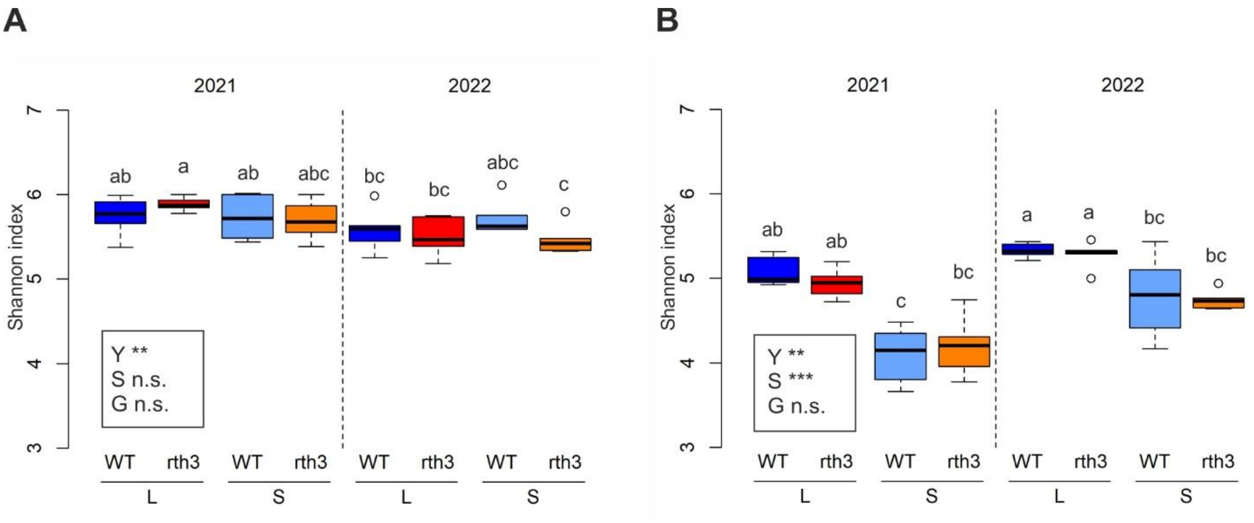

FIG. S8. Shannon Index of the 16S rRNA-based (A) and 1 – aminocyclopropane – 1 – carboxylate deaminase (*acdS*$^+$)-based (B) microbial community structure in the rhizosphere of maize between the years 2021 (moist) and 2022 (dry). B73 maize wild type (WT) and root hair deficient mutant 3 (*rth3*), grown on the substrates loam (L) and sand (S). Significances were determined using Kruskal-Wallis Test, followed by Dunn‘s Test. Asterisk ** and *** indicate $p < 0.01$ and < 0.001, respectively; whereas n.s. represents non-significant differences for the factors year (Y), substrate (S) and genotype (G) (n = 6, except for WT grown in sand in 2021 (n=5)). Letters indicate significant differences between treatments.

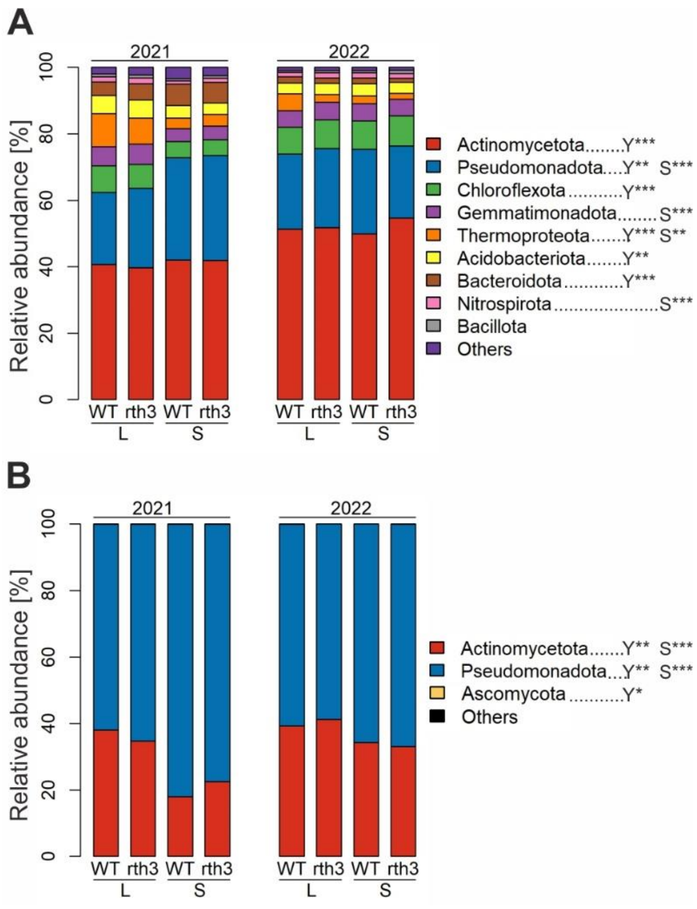

FIG. S9. Relative abundance of the 16S rRNA-based (A) and 1 – aminocyclopropane – 1 – carboxylate deaminase (*acdS*$^{+}$)-based (B) microbial community structure at phylum level in the rhizosphere of maize roots between the years 2021 (moist) and 2022 (dry). B73 maize wild type (WT) and root hair deficient mutant 3 (*rth3*), grown on the substrates loam (L) and sand (S). Asterisks represent statistical significances of the factor year (Y), substrate (S) and genotype (G) with ** < 0.01, *** < 0.001 and n.s. for not significant (Kruskal-Wallis Test, followed by Dunn's Test, n = 6, except for WT grown in sand in 2021 (n=5)).

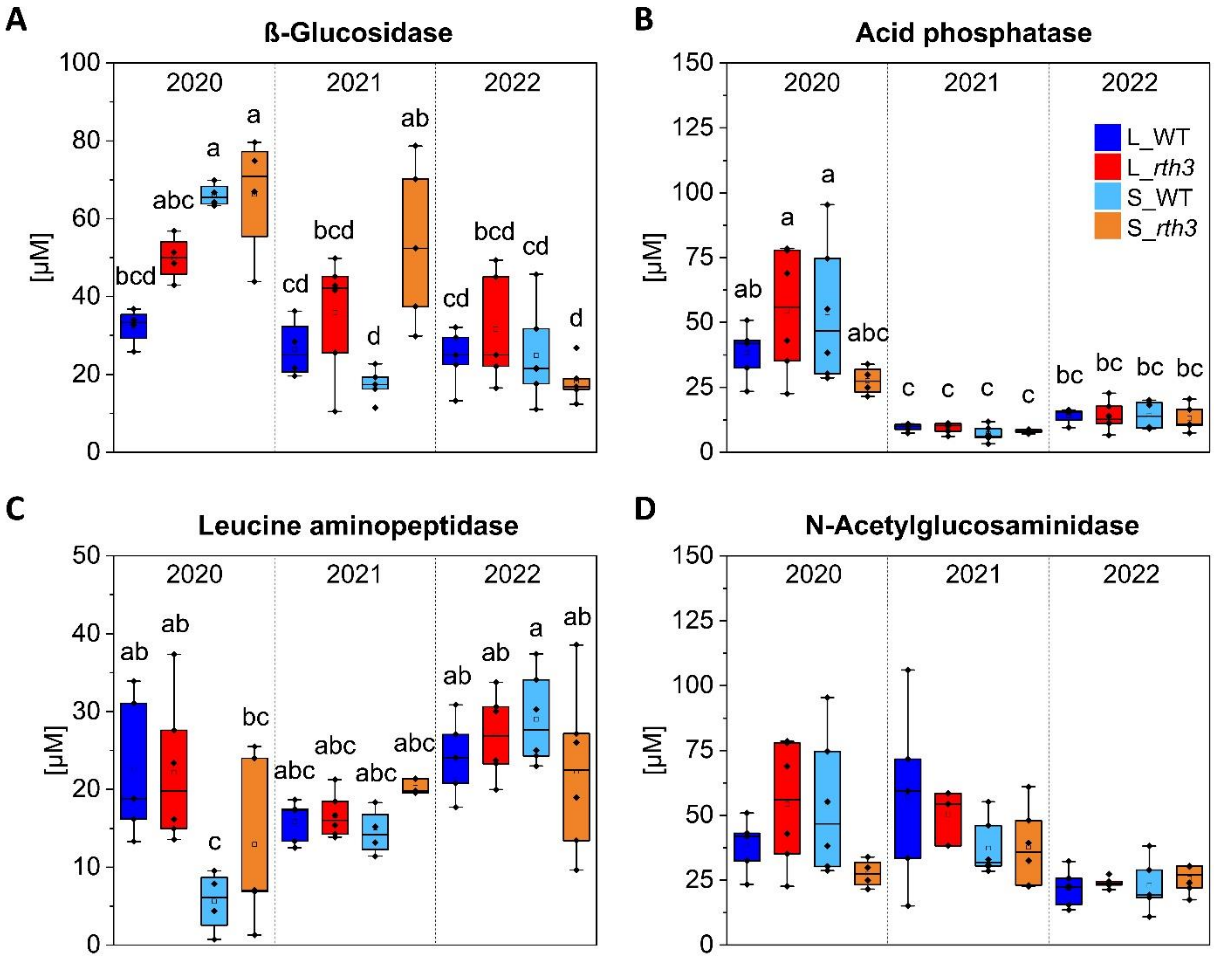

FIG. S10. Affinity constant (K_m) of enzymes (**a-d**) in rhizosphere collected under B73 maize wild type (WT) and root hair deficient mutant 3 (*rth3*) grown on the substrates loam (L) and sand (S), in three years with different precipitation levels (2020 - dry, 2021 - moist, 2022 - dry). Different letters indicate significant differences according to a Generalized Linear Model and Holm-Bonferroni method ($p < 0.05$), n= 3-6, as represented by data points. Letters indicate significant differences between treatments.
