# Supplementary material for: Responses of maize roots, rhizosphere enzyme kinetics and prokaryote diversity to alternating precipitation: insights from a three-year field study
Source: Ann Bot. 2025 Aug 6;136(5-6):1081–99. doi: 10.1093/aob/mcaf180 (PMC12682858; doi:10.1093/aob/mcaf180)
Supplement: mcaf180_Supplementary_Data [file mcaf180_supplementary_data.zip › Supplementary_Material_Table_of_Content.pdf]

Responses of Maize Roots, Rhizosphere Enzyme Kinetics, and Prokaryote Diversity to Alternating Precipitation: Insights from a Three-Year Field Study

SUPPLEMENTARY DATA – TABLE OF CONTENT

Item	Contribution
**FIG. S1**	Temperature at the weather station in Bad Lauchstädt, Germany and soil volumetric water content on sampling days
**FIG. S2**	Volumetric water content on sampling days
**FIG. S3**	Precipitation at the weather station in Bad Lauchstädt, Germany, in the years 2018 to 2023 and at growth stage BBCH14 and BBCH19 in the years 2020, 2021 and 2022
**FIG. S4**	Volumetric water content from sowing until harvest
**FIG. S5**	Gene ontology terms enriched in maize root up- and down-regulated genes between 2022 and 2020
**FIG. S6**	Gene ontology terms enriched in maize root up- and down-regulated genes between the two dry years and a moist year for the two substrates
**FIG. S7**	Gene ontology terms enriched in maize root up- and down-regulated genes between the two dry years for the two substrates
**FIG. S8**	Shannon Index of rhizosphere prokaryote structure
**FIG. S9**	Relative abundance of rhizosphere prokaryote structure
**FIG. S10**	Affinity constant (K_m) of rhizosphere enzymes
**TABLE S1**	Differentially expressed maize root genes related to drought and heat stress
**TABLE S2**	Differentially expressed maize root genes related to mineral element uptake
**TABLE S3**	Differentially expressed maize root genes related to immunity and defense
**TABLE S4**	Differentially expressed maize root genes related to exudation and secondary metabolism
**TABLE S5**	Differentially expressed maize root genes related to cell wall structure
**TABLE S6**	Raw and filtered values of maximum enzymatic rate (V_{max}) and affinity constant (K_m) of rhizosphere enzymes
**TABLE S7**	Nutrient concentrations in the youngest unfolded leaf of maize plants
**TABLE S8**	Genes associated with the rth3 (root hair defective 3) mutation between B73 maize wild type (WT) and *rth3* mutant roots
**TABLE S9**	Permutational analysis of variance (PERMANOVA) of root gene expression levels for the two substrates
