# Supplementary material for: Responses of maize roots, rhizosphere enzyme kinetics and prokaryote diversity to alternating precipitation: insights from a three-year field study
Source: Ann Bot. 2025 Aug 6;136(5-6):1081–99. doi: 10.1093/aob/mcaf180 (PMC12682858; doi:10.1093/aob/mcaf180)
Supplement: mcaf180_Supplementary_Data [file mcaf180_supplementary_data.zip › Supplementary_Tables_S1-S9.pdf]

Responses of Maize Roots, Rhizosphere Enzyme Kinetics, and Prokaryote Diversity to Alternating Precipitation: Insights from a Three-Year Field Study

SUPPLEMENTARY DATA – TABLES

TABLE S1. *Differentially expressed genes in maize roots related to drought and heat stress. Two maize genotypes, the B73 wild type (WT) and root hair deficient mutant 3 (rth3) were grown on the substrates loam and sand in three years with different precipitation levels (2020 – dry, 2021 – moist, 2022 – dry). Genes were selected from a list of differentially expressed genes comparing: 2020 vs. 2021, 2022 vs. 2021 and 2022 vs.2020. Filtering was done by searching for the following words: ABA, abscisic, aquaporin, AREB, dehydration, dehydrin, DREB, heat, embryogenesis, EREB, ERF, ethylene, LEA, MYB, NAC, PIP, TIP, WRKY. Note that the first part of the list considers differences in gene expression between the years 2020 and 2021, the second 2022 and 2021, and the third 2022 vs 2020. In 2020 n = 3 for plants grown on loam and n = 4 for plants grown in sand; in 2021 & 2022 n = 6, except for rth3 mutant grown in loam (n = 5). Positive and negative Log2fold indicates up-and downregulation, respectively. Common abbreviations used in gene names include: ABA – Abscisic Acid; ERF – Ethylene Responsive Factor; PIP - Plasma Membrane Intrinsic Protein; TIP - Tonoplast Intrinsic Protein; MYB - Myeloblastosis.*

**Gene ID**	**Annotation**	**Log2fold**
2020 vs. 2021		
Zm00001eb418610	Heat shock protein (HSP) 90 1	3.545082
Zm00001eb184890	MYB domain protein 79	3.067739
Zm00001eb124950	17.6 kDa class I Heat shock protein 1 related	2.33202
Zm00001eb124940	Small Heat shock protein HSP20 family	1.634051
Zm00001eb124960	Small Heat shock protein HSP20 family	1.47789
Zm00001eb011900	Small Heat shock protein HSP20 family	1.384414
Zm00001eb403580	NAC containing protein 76 related	1.233572
Zm00001eb011880	Small Heat shock protein HSP20 family	1.214584
Zm00001eb106790	MYB like DNA binding protein	1.150862
Zm00001eb047760	Heat shock transcription factor	1.053541
Zm00001eb074730	AP2/ERF domain	-1.03002
Zm00001eb026680	Small Heat shock protein HSP20 family	-1.15941
Zm00001eb052730	Small Heat shock protein HSP20 family	-1.18704
Zm00001eb026690	Small Heat shock protein HSP20 family	-1.20135

Zm00001eb067980	AP2 like factor	-1.2607
Zm00001eb100410	Heat shock transcription factor	-1.26986
Zm00001eb160630	Small Heat shock protein HSP20 family	-1.32929
Zm00001eb384760	Heat shock transcription factor	-1.59082
Zm00001eb167200	Late embryogenesis abundant protein (LEA)	-1.63792
Zm00001eb201150	MYB like DNA binding protein	-1.65859
Zm00001eb026540	Small Heat shock protein HSP20 family	-1.70875
Zm00001eb107500	Ethylene responsive transcription factor CRF3 related	-1.91523
Zm00001eb172240	SANT/MYB domain	-2.42817
2022 vs. 2021		
Zm00001eb005250	Late embryogenesis abundant plants LEA related	8.165009
Zm00001eb428350	Late embryogenesis abundant (LEA) group 1	7.919819
Zm00001eb418610	Heat shock protein 90	6.55809
Zm00001eb308610	Late embryogenesis abundant protein 4 5	6.128321
Zm00001eb294480	Late embryogenesis abundant domain containing protein	5.863937
Zm00001eb285360	Dehydrin	5.254484
Zm00001eb414650	Late embryogenesis abundant (LEA) group 1	4.938099
Zm00001eb004470	LEA protein related	4.234693
Zm00001eb182890	Ethylene insensitive protein 3 (EIN3)	4.185067
Zm00001eb011930	Small heat shock protein	3.973986
Zm00001eb056980	Heat shock transcription factor	3.617352
Zm00001eb155430	Late embryogenesis abundant plants LEA related	3.581326
Zm00001eb397600	Heat shock protein 70 family	3.469956
Zm00001eb337690	Class II heat shock protein related	3.3186
Zm00001eb422470	AP2/ERF domain	3.277005
Zm00001eb124960	Small heat shock protein HSP20	3.236977
Zm00001eb076690	Aquaporin TIP3 1 related	3.228637
Zm00001eb176720	ABA 8' hydroxylase	3.152068
Zm00001eb044880	Aquaporin transporter	3.120844
Zm00001eb368000	Heat shock protein 1/8	3.067871
Zm00001eb257130	Class III heat shock protein	2.897909
Zm00001eb399810	NAC domain containing protein 2	2.877110
Zm00001eb148420	Heat shock 70kDa protein 1/8	2.377474
Zm00001eb106790	MYB transcription factor related	2.363097
Zm00001eb428800	Heat shock transcription factor	2.225391
Zm00001eb342130	Late embryogenesis abundant protein LEA 14	2.221707
Zm00001eb184890	MYB domain protein 79	2.21669
Zm00001eb010530	Small heat shock protein	2.18651
Zm00001eb250120	Dehydrin COR47 related	2.090709
Zm00001eb131500	ABA/WDS induced protein	2.216686
Zm00001eb187010	Dehydrin COR47 related	1.742444
Zm00001eb011880	Small heat shock protein	1.604276
Zm00001eb011900	Small heat shock protein	1.538736
Zm00001eb242480	Heat shock transcription factor A 3	1.457922

Zm00001eb047760	Heat shock transcription factor	1.429525
Zm00001eb235800	Small heat shock protein HSP20	1.316465
Zm00001eb234870	EREBP like factor	1.27780
Zm00001eb009170	Heat shock transcription factor	1.252047
Zm00001eb075450	Heat shock chaperone binding	1.078226
Zm00001eb232010	Heat shock protein HSP90	1.067632
Zm00001eb067980	AP2 like factor	1.067503
Zm00001eb044970	Late embryogenesis abundant LEA related	-1.03062
Zm00001eb074730	AP2/ERF domain	-1.05508
Zm00001eb119080	Late embryogenesis abundant protein (LEA)	-1.07893
Zm00001eb204180	Abscisic acid receptor PYR/PYL family	-1.132777
Zm00001eb249980	Late embryogenesis abundant protein (LEA)	-1.20701
Zm00001eb077130	Aquaporin PIP	-1.23831
Zm00001eb096680	Aquaporin PIP	-1.39125
Zm00001eb190950	Aquaporin PIP 1-5	-1.42307
Zm00001eb247760	Aquaporin PIP	-1.46993
Zm00001eb186570	Aquaporin transporter TIP 2 3 related	-1.4962
Zm00001eb223910	Aquaporin transporter	-1.58573
Zm00001eb026680	Small heat shock protein HSP20	-1.66467
Zm00001eb201150	MYB transcription factor related	-1.738754
Zm00001eb306400	Aquaporin PIP	-1.75715
Zm00001eb416440	Aquaporin transporter	-1.76024
Zm00001eb337830	Late embryogenesis abundant protein (LEA)	-1.81762
Zm00001eb365110	Late embryogenesis abundant protein (LEA)	-1.8501
Zm00001eb138020	Late embryogenesis abundant protein (LEA)	-1.88325
Zm00001eb119200	Aquaporin transporter	-2.18823
Zm00001eb172240	SANT/MYB domain	-2.222567
Zm00001eb429750	Aquaporin TIP2 3 related	-2.2997
Zm00001eb185300	Aquaporin PIP	-2.40521
Zm00001eb167200	Late embryogenesis abundant protein (LEA)	-2.57479
Zm00001eb358700	Heat shock transcription factor C 1	-2.83707
Zm00001eb429760	Aquaporin TIP	-2.83733
Zm00001eb095450	Late embryogenesis abundant protein (LEA)	-3.05075
Zm00001eb107500	Ethylene responsive transcription factor CRF3 related	-3.310074
Zm00001eb279660	Aquaporin transporter	-3.34155
Zm00001eb239170	Aquaporin transporter NIP1 related	-3.35447
Zm00001eb074660	Aquaporin transporter TIP2 3	-3.7943
Zm00001eb096620	Aquaporin PIP	-4.08682
2022 vs. 2020		
Zm00001eb005250	Late embryogenesis abundant plants LEA related	10.06574
Zm00001eb414650	Late embryogenesis abundant (LEA) group 1	6.843284
Zm00001eb428350	Late embryogenesis abundant (LEA) group 1	6.625587
Zm00001eb308610	Late embryogenesis abundant protein 4 5	6.05256
Zm00001eb294480	Late embryogenesis abundant domain containing protein	5.146114

Zm00001eb285360	Dehydrin	4.522309
Zm00001eb155430	Late embryogenesis abundant plants LEA related	4.165877
Zm00001eb399810	NAC domain containing protein 2	3.247319
Zm00001eb004470	LEA protein related	3.030218
Zm00001eb384760	Heat shock transcription factor	2.757636
Zm00001eb397600	Heat shock protein 70 family	2.738035
Zm00001eb423300	Small heat shock protein HSP20 family	2.4009
Zm00001eb067980	AP2 like factor 2	2.34068
Zm00001eb239380	Heat shock transcription factor	2.219806
Zm00001eb342130	Late embryogenesis abundant protein, LEA 14	2.155756
Zm00001eb250120	Dehydrin COR47 related	1.812752
Zm00001eb131500	ABA/WDS induced protein	1.755653
Zm00001eb314350	Small heat shock protein HSP20 family	1.753934
Zm00001eb136760	MYB like DNA binding protein	1.650652
Zm00001eb085540	Heat shock protein DnaJ, cysteine rich domain	1.550631
Zm00001eb187010	Dehydrin COR47 related	1.490988
Zm00001eb149580	WRKY DNA binding domain	1.424544
Zm00001eb242480	Heat shock transcription factor A 3	1.267884
Zm00001eb190950	Aquaporin PIP 1-5	-1.04257
Zm00001eb352720	Small heat shock protein HSP20 family	-1.14979
Zm00001eb328600	MYB like DNA binding protein	-1.193296
Zm00001eb247760	Aquaporin PIP	-1.3289
Zm00001eb339810	MYB like DNA binding protein	-1.367125
Zm00001eb257030	Ethylene responsive transcription factor ERF018	-1.470592
Zm00001eb337830	Late embryogenesis abundant protein	-1.47092
Zm00001eb415280	Late embryogenesis abundant protein (LEA)	-1.47655
Zm00001eb323160	Late embryogenesis abundant protein, LEA 14	-1.65364
Zm00001eb223910	Aquaporin TRANSPORTER	-1.65504
Zm00001eb185300	Aquaporin PIP	-1.88195
Zm00001eb291140	MYB like DNA binding domain	-2.179113
Zm00001eb429750	Aquaporin TIP2 3 related	-2.32799
Zm00001eb416440	Aquaporin transporter	-2.46639
Zm00001eb124940	Small heat shock protein HSP20 family	-2.78983
Zm00001eb358700	Heat shock transcription factor	-3.12899
Zm00001eb239170	Aquaporin transporter	-3.23784
Zm00001eb096620	Aquaporin PIP	-3.52345
Zm00001eb074660	Aquaporin TIP2 3 related	-3.56397

Table S2. *Differentially expressed genes in maize roots related to mineral element uptake. Two maize genotypes, the B73 wild type (WT) and root hair deficient mutant 3 (rth3) were grown on the substrates loam and sand in three years with different precipitation levels (2020 – dry, 2021 – moist, 2022 – dry). Genes were selected from a list of differentially expressed genes comparing: 2020 vs. 2021, 2022 vs. 2021 and 2022 vs.2020. Filtering was done by searching for the following words: transport, channel, uptake, nicotianamin, efflux, carrier, export, import, pump, exchanger. Note that the first part of the list considers differences in gene expression between the years 2020 and 2021, the second 2022 and 2021, and the third 2022 vs 2020. In 2020 n = 3 for plants grown on loam and n = 4 for plants grown in sand; in 2021 & 2022 n = 6, except for rth3 mutant grown in loam (n = 5). Positive and negative Log2fold indicates up-and downregulation, respectively. Common abbreviations used in gene names include: ATP - Adenosine Triphosphate; ATOX - Antioxidant Protein; ABC - ATP-Binding Cassette.*

Gene ID	Annotation	Log2fold
**2020 vs. 2021**		
Zm00001eb418780	Nucelobase ascorbate transporter 11	3.246416
Zm00001eb252970	Chloride channel protein CLC F	3.167926
Zm00001eb305560	Copper transport protein ATOX1 related	2.807242
Zm00001eb105270	F type H+ transporting ATPase subunit beta (ATPF1B, atpD)	2.355417
Zm00001eb087740	Phosphate transporting ATPase / ABC phosphate transporter	2.27951
Zm00001eb308910	Maltose transporting ATPase	2.246402
Zm00001eb087730	Phosphate transporting ATPase / ABC phosphate transporter	1.83754
Zm00001eb317200	Cation transporting P type ATPase, N	1.629375
Zm00001eb429050	Sodium/hydrogen exchanger	1.493707
Zm00001eb038750	Cation transport protein CHAC related	1.306577
Zm00001eb095410	Sulfate transporter 4 (SULTR4)	1.145427
Zm00001eb165450	Magnesium/proton exchanger (MHX)	1.035874
Zm00001eb026490	Amino acid transporter	-1.12516
Zm00001eb279680	Amino acid transporter	-1.1407
Zm00001eb010460	Amino acid transporter	-1.16529
Zm00001eb080220	Copper transport protein ATOX1 related	-1.19107
Zm00001eb246140	High affinity nitrate transporter accessory (NAR2)	-1.19487
Zm00001eb214890	Ammonium transporter	-1.21072
Zm00001eb357860	Amino acid transporters	-1.29978
Zm00001eb222610	Copper transport protein ATOX1 related	-1.30783
Zm00001eb080210	Copper transport protein ATOX1 related	-1.34652
Zm00001eb424580	Copper transport protein ATOX1 related	-1.44278
Zm00001eb359320	Voltage and ligand gated potassium channel	-1.54599
Zm00001eb080230	Copper transport protein ATOX1 related	-1.5507
Zm00001eb288010	Oligopeptide transporter related	-1.6121
Zm00001eb311300	Sugar transporter/spinster transmembrane protein	-1.63354
Zm00001eb016620	Copper transport protein ATOX1 related	-1.68415
Zm00001eb081160	Sugar/inositol transporter	-1.7188

Zm00001eb401500	Purine nucleobase transmembrane transport (PUNUT)	-1.73895
Zm00001eb127390	Copper transport protein ATOX1 related	-1.90204
Zm00001eb366570	Oligopeptide transporter related	-3.43929
**2022 vs. 2021**		
Zm00001eb249190	Sodium dependent phosphate transporter	2.807073
Zm00001eb048930	Osmotic stress potassium transporter	2.581111
Zm00001eb238560	Sodium/calcium exchanger membrane region	2.350119
Zm00001eb412870	Calcium transporting ATPase	2.182349
Zm00001eb222960	Calcium transporting ATPase 12, plasma membrane type	1.853659
Zm00001eb169870	Potassium channel, subfamily K	1.809425
Zm00001eb412940	Lysine histidine transporter 1 related	1.722208
Zm00001eb079530	High affinity nitrate transporter	1.708091
Zm00001eb395430	Potassium channel, voltage dependent, tandem inactivation domain	1.668801
Zm00001eb048950	Osmotic stress potassium transporter	1.642871
Zm00001eb261480	Lysine histidine transporter 1 related	1.546684
Zm00001eb108730	Copper transport protein ATOX1 related	1.462359
Zm00001eb322290	Organic solute transporter related	1.306868
Zm00001eb161780	ATP binding cassette transporter	1.162198
Zm00001eb042570	Phospholipid transporting ATPase 8 related	1.048687
Zm00001eb338370	Sodium/calcium exchanger membrane region	1.03568
Zm00001eb314770	Mitochondrial phosphate carrier protein 2	1.033202
Zm00001eb038300	Sodium channel modifier 1	1.026277
Zm00001eb158530	Molybdate transporter 2	-1.10223
Zm00001eb334940	Copper transport protein ATOX1 related	-1.10804
Zm00001eb080210	Copper transport protein ATOX1 related	-1.15654
Zm00001eb366300	Amino acid transporter	-1.16201
Zm00001eb031850	Cation/H(+) antiporter 19	-1.20557
Zm00001eb289290	Oligopeptide transporter related	-1.21864
Zm00001eb034200	Calcium transporting ATPase 2, plasma membrane related	-1.28522
Zm00001eb047550	Na+/Ca^{2}+ K+ independent exchanger	-1.28662
Zm00001eb394840	Copper transport protein ATOX1 related	-1.29881
Zm00001eb084680	Copper transport protein ATOX1 related	-1.31737
Zm00001eb288800	Amino acid transporter	-1.31978
Zm00001eb071010	Zinc/Iron transporter	-1.32833
Zm00001eb201000	Solute carrier family 31 copper transporter	-1.36105
Zm00001eb176380	Osmotic stress potassium transporter	-1.41144
Zm00001eb178270	Amino acid transporter	-1.4128
Zm00001eb424580	Copper transport protein ATOX1 related	-1.42167
Zm00001eb258520	Phosphate transporter PHO1	-1.4616
Zm00001eb197320	Amino acid transporter	-1.48297
Zm00001eb211600	Oligopeptide transporter related	-1.48929
Zm00001eb018250	Amino acid transporter	-1.51538
Zm00001eb009150	Sugar transporter/spinster transmembrane protein	-1.53298
Zm00001eb428870	Aluminium activated malate transporter (ALMT)	-1.53579

Zm00001eb401500	Purine nucleobase transmembrane transport (PUNUT)	-1.5568
Zm00001eb299500	Amino acid transporter	-1.63911
Zm00001eb275630	Potassium transporter 6	-1.6521
Zm00001eb008160	Sulfate transporter 1, high affinity (SULTR1)	-1.67269
Zm00001eb031840	Cation/H(+) antiporter 19	-1.68037
Zm00001eb016620	Copper transport protein ATOX1 related	-1.68414
Zm00001eb127390	Copper transport protein ATOX1 related	-1.68691
Zm00001eb290160	Zinc/Iron transporter	-1.70867
Zm00001eb080230	Copper transport protein ATOX1 related	-1.71298
Zm00001eb279680	Amino acid transporter	-1.76307
Zm00001eb119760	Bidirectional sugar transporter SWEET3	-1.77099
Zm00001eb292010	Voltage and ligand gated potassium channel	-1.79427
Zm00001eb311300	Sugar transporter/spinster transmembrane protein	-1.8232
Zm00001eb120240	Cationic amino acid transporter 5	-1.84827
Zm00001eb214890	Ammonium transporter	-1.9238
Zm00001eb052440	Zinc transporter 7	-1.96441
Zm00001eb349050	Amino acid transporter	-1.96567
Zm00001eb118350	Osmotic stress potassium transporter	-2.02143
Zm00001eb383670	Copper transport protein ATOX1 related	-2.02249
Zm00001eb038730	Phosphate transporting ATPase / ABC phosphate transporter	-2.09268
Zm00001eb081780	Voltage and ligand gated potassium channel	-2.11727
Zm00001eb074360	Cu+ exporting ATPase (copA, ATP7)	-2.12223
Zm00001eb430910	Copper transport protein ATOX1 related	-2.13868
Zm00001eb154590	Sulfate transporter	-2.14899
Zm00001eb014680	Nicotianamine synthase (2.5.1.43)	-2.33501
Zm00001eb087740	Phosphate transporting ATPase / ABC phosphate transporter	-2.36951
Zm00001eb190060	Calcium activated chloride channel regulator	-2.48857
Zm00001eb014700	Nicotianamine synthase (2.5.1.43)	-2.49021
Zm00001eb222260	Calcium activated chloride channel regulator	-2.76846
Zm00001eb080050	Amino acid transporter	-2.81005
Zm00001eb396110	Nicotianamine synthase (2.5.1.43)	-2.97256
Zm00001eb139810	Zinc/Iron transporter	-3.02555
Zm00001eb087730	Phosphate transporting ATPase / ABC phosphate transporter	-3.22486
Zm00001eb406990	Basic amino acid/polyamine antiporter, APA family (TC.APA)	-3.33954
Zm00001eb227000	Na+/K+ transporter	-4.3647
Zm00001eb004100	Inorganic phosphate transporter (PHO84)	-6.64971
**2022 vs. 2020**		
Zm00001eb366570	Oligopeptide transporter related	3.412799
Zm00001eb048940	Osmotic stress potassium transporter	2.656477
Zm00001eb025860	Oligopeptide transporter related	2.344488
Zm00001eb048930	Osmotic stress potassium transporter	2.172158
Zm00001eb057860	Cyclic nucleotide gated ion channel 2	1.906126
Zm00001eb080220	Copper transport protein ATOX1 related	1.753139
Zm00001eb381570	Sodium/hydrogen exchanger 4	1.721555

Zm00001eb324230	transporter B0361.11 related	1.7205
Zm00001eb395430	Potassium channel	1.710169
Zm00001eb261480	Lysine histidine transporter 1 related	1.70768
Zm00001eb160640	Voltage dependent anion selective channel	1.64198
Zm00001eb073510	Predicted transporter/transmembrane protein	1.484415
Zm00001eb254200	Nucleobase ascorbate transporter 5 related	1.421702
Zm00001eb403950	Solute carrier family 35	1.419308
Zm00001eb178810	Sulfate transporter 1.1 related	1.36159
Zm00001eb322880	ABC transporter G family member 34 related	1.307084
Zm00001eb245460	Copper transport protein ATOX1 related	1.161695
Zm00001eb074380	Cu+ exporting ATPase	-1.08348
Zm00001eb197320	Amino acid transporter	-1.1069
Zm00001eb022580	Predicted transporter ADD1	-1.16513
Zm00001eb158530	Molybdate transporter 2	-1.21419
Zm00001eb047550	Na+/Ca^{2}+ K+ independent exhanger	-1.23012
Zm00001eb118730	Voltage dependent anion channel (SLAC1)	-1.27218
Zm00001eb289290	Protein NRT1/ PTR family 5.10 related	-1.38424
Zm00001eb249990	Nucleobase:cation symporter 1	-1.43787
Zm00001eb081780	Voltage and ligand gated potassium channel	-1.44326
Zm00001eb018250	Amino acid transporter	-1.47143
Zm00001eb046210	Oligopeptide transporter related	-1.51086
Zm00001eb074360	Cu+ exporting ATPase	-1.5616
Zm00001eb052440	Zinc transporter 7	-1.5692
Zm00001eb071010	Zinc/Iron transporter	-1.57491
Zm00001eb126380	Phosphate transporter PHO1 homolog 1	-1.58048
Zm00001eb009150	Sugar transporter/spinster transmembrane protein	-1.6084
Zm00001eb275630	Potassium transporter 6	-1.61014
Zm00001eb428870	Aluminum activated malate transporter (ALMT)	-1.6344
Zm00001eb172740	ABC transporter like 1	-1.81052
Zm00001eb359490	Phosphate transporting ATPase	-1.83528
Zm00001eb349050	Amino acid transporter	-1.85956
Zm00001eb290160	Zinc/Iron transporter	-1.92825
Zm00001eb172720	ABC transporter transmembrane region	-2.01148
Zm00001eb292010	Voltage and ligand gated potassium channel	-2.01493
Zm00001eb080770	Transmembrane amino acid transporter	-2.10981
Zm00001eb154590	Sulfate transporter 3.5 related	-2.4142
Zm00001eb406990	Basic amino acid/polyamine antiporter	-2.55575
Zm00001eb252970	Chloride channel protein CLC F	-2.5818
Zm00001eb137340	Voltage dependent anion channel	-2.63421
Zm00001eb038730	Phosphate transporting ATPase	-2.75968
Zm00001eb139810	Zinc/Iron transporter	-2.81315
Zm00001eb014680	Nicotianamine synthase (2.5.1.43)	-2.99216
Zm00001eb014700	Nicotianamine synthase (2.5.1.43)	-3.30612
Zm00001eb418780	Nucleobase ascorbate transporter 11	-3.73946

Zm00001eb396230	Nicotianamine synthase (2.5.1.43)	-4.41923
Zm00001eb087740	Phosphate transporting ATPase	-4.63897
Zm00001eb227000	Na+/K+ transporter	-4.70067
Zm00001eb087730	Phosphate transporting ATPase	-5.05391

TABLE S3. *Differentially expressed genes in maize roots related to immunity and defense. Two maize genotypes, the B73 wild type (WT) and root hair deficient mutant 3 (rth3) were grown on the substrates loam and sand in three years with different precipitation levels (2020 – dry, 2021 – moist, 2022 – dry). Genes were selected from a list of differentially expressed genes comparing: 2020 vs. 2021, 2022 vs. 2021 and 2022 vs.2020. Filtering was done by searching for the following words: pathogenesis, flagellin, cerk, elongation, efr, lysm, wak, rlk, thaumatin, chitinase. Note that the first part of the list considers differences in gene expression between the years 2020 and 2021, the second 2022 and 2021, and the third 2022 vs 2020. In 2020 n = 3 for plants grown on loam and n = 4 for plants grown in sand; in 2021 & 2022 n = 6, except for rth3 mutant grown in loam (n = 5). Positive and negative Log2fold indicates up-and downregulation, respectively.*

Gene ID	Annotation	Log2fold
2020 vs. 2021		
Zm00001eb399560	LysM domain (LysM)	-1.107189
Zm00001eb398830	Thaumatin family	-1.226303
Zm00001eb226040	Thaumatin family	-1.316897
Zm00001eb192760	Pathogenesis related 1 protein	-1.404325
Zm00001eb217970	Thaumatin family	-1.433563
Zm00001eb257320	Pathogenesis related 1 protein	-1.511971
Zm00001eb332120	Thaumatin family	-1.524364
Zm00001eb164410	Thaumatin family	-1.744138
Zm00001eb102170	Thaumatin family	-1.86958
2022 vs. 2021		
Zm00001eb010450	Thaumatin family (Thaumatin)	2.308701
Zm00001eb150050	Thaumatin family (Thaumatin)	2.000796
Zm00001eb299340	Pathogenesis related protein 1 (PR1)	1.714692
Zm00001eb384600	Thaumatin family (Thaumatin)	1.222819
Zm00001eb174700	Thaumatin family (Thaumatin)	1.200848
Zm00001eb303120	Pathogenesis related thaumatin superfamily protein	-1.026269
Zm00001eb202030	Thaumatin family (Thaumatin) Protein kinase domain	-1.033496
2022 vs. 2020		
Zm00001eb010450	Thaumatin family	2.794711
Zm00001eb052520	Thaumatin family	2.333233
Zm00001eb226040	Thaumatin family	2.013088
Zm00001eb299340	Pathogenesis related protein 1 (PR1)	1.999115
Zm00001eb299370	Pathogenesis related protein 1 (PR1)	1.930371
Zm00001eb048140	Thaumatin family	1.714238
Zm00001eb217970	Thaumatin family	1.340747
Zm00001eb202030	Thaumatin family	-1.308981

TABLE S4. *Differentially expressed genes in maize roots related to exudation and secondary metabolism. Two maize genotypes, the B73 wild type (WT) and root hair deficient mutant 3 (rth3) were grown on the substrates loam and sand in three years with different precipitation levels (2020 – dry, 2021 – moist, 2022 – dry). Genes were selected from a list of differentially expressed genes comparing: 2020 vs. 2021, 2022 vs. 2021 and 2022 vs.2020. Filtering was done by searching for the following words: terpen, benzo, phenyl, neomenthol, alkaloid, flavon, saponin, chalcon, squalen, methyltransferase, anthocyan, jasmonat, SWEET, MATE, ALMT, malate, extrusion, DIBOA, salicylic, coumaric, cinnamic, gallic, ferulic. Note that the first part of the list considers differences in gene expression between the years 2020 and 2021, the second 2022 and 2021, and the third 2022 vs 2020. In 2020 n = 3 for plants grown on loam and n = 4 for plants grown in sand; in 2021 & 2022 n = 6, except for rth3 mutant grown in loam (n = 5). Positive and negative Log2fold indicates up-and downregulation, respectively. Common abbreviations used in gene names include: MATE - Multidrug And Toxic compound Extrusion transporter; ZIM - Zinc-finger Inflorescence Meristem.*

Gene ID	Annotation	Log2fold
2020 vs. 2021		
Zm00001eb372870	Adenine specific methyltransferase, domain 2	3.926939
Zm00001eb022840	Methyltransferase	2.410864
Zm00001eb371800	Anthranilate O methyltransferase	1.844192
Zm00001eb202870	MATE efflux family protein	1.828825
Zm00001eb048870	MATE efflux family protein	1.470015
Zm00001eb273480	Methyltransferase PMT9 related	1.159794
Zm00001eb058110	Isoflavone 2' hydroxylase / Isoflavone 2' monooxygenase	-1.20193
Zm00001eb048860	MATE efflux family protein	-1.27512
Zm00001eb081850	Methyltransferase	-1.31591
Zm00001eb058050	Isoflavone 2' hydroxylase / Isoflavone 2' monooxygenase	-1.32441
Zm00001eb058060	Isoflavone 2' hydroxylase / Isoflavone 2' monooxygenase	-1.46016
Zm00001eb230520	Histone lysine methyltransferase ATXR5	-1.52409
Zm00001eb294690	Phosphoethanolamine N methyltransferase (E2.1.1.103, NMT)	-1.63419
Zm00001eb260260	Methyltransferase related	-1.68861
Zm00001eb260740	Methyltransferase	-1.70152
Zm00001eb138830	Methyltransferase PMT24 related	-1.73207
Zm00001eb102910	Methyltransferase domain	-1.97057
Zm00001eb145350	Methyltransferase	-2.5189
Zm00001eb198150	Trans resveratrol di O methyltransferase / Resveratrol O	-2.90839
2022 vs. 2021		
Zm00001eb098950	Flavonol 3 O glucosyltransferase	1.998354
Zm00001eb075660	Neomenthol dehydrogenase / Monoterpenoid dehydrogenase	1.918803
Zm00001eb311610	Flavanone 7 O beta glucosyltransferase	1.815241
Zm00001eb058060	Isoflavone 2' hydroxylase / Isoflavone 2' monooxygenase	1.776035
Zm00001eb373710	Chalcone isomerase	1.424538
Zm00001eb058100	Isoflavone 2' hydroxylase / Isoflavone 2' monooxygenase	1.316755

Zm00001eb314010	Jasmonate ZIM domain containing protein (JAZ)	1.210325
Zm00001eb212500	Sterol 24 C methyltransferase	1.205296
Zm00001eb022840	Methyltransferase	1.188496
Zm00001eb362400	Predicted methyltransferase (contains a set domain)	1.182183
Zm00001eb005990	Jasmonate ZIM domain containing protein (JAZ)	1.178361
Zm00001eb084980	Jasmonate ZIM domain containing protein (JAZ)	1.042094
Zm00001eb399940	Homocysteine S methyltransferase	1.021835
Zm00001eb077230	Phenylalanine/tyrosine ammonia lyase (PTAL)	-1.01353
Zm00001eb017620	Flavonoid 3',5' hydroxylase / F3'5'H	-1.1466
Zm00001eb168290	O Methyltransferase	-1.16991
Zm00001eb117370	Flavonoid 3' monooxygenase / Flavonoid 3' hydroxylase	-1.31036
Zm00001eb164860	2.4 dihydroxy 1.4 benzoxazin	-1.33915
Zm00001eb102940	Methyltransferase domain	-1.49817
Zm00001eb211050	Multidrug resistance protein (MATE efflux family protein)	-1.5288
Zm00001eb428870	Aluminum activated malate transporter (ALMT)	-1.53579
Zm00001eb138830	Methyltransferase PMT24 related	-1.57421
Zm00001eb254700	Methyltransferase // pectin methyltransferase QUA2 related	-1.62451
Zm00001eb292850	O Methyltransferase	-1.62657
Zm00001eb327700	Phenylalanine ammonia lyase	-1.7743
Zm00001eb102910	Methyltransferase domain	-1.82982
Zm00001eb212360	(+) neomenthol dehydrogenase / Monoterpenoid dehydrogenase	-1.84609
Zm00001eb394430	2 hydroxyisoflavanone synthase / Isoflavonoid synthase	-1.8848
Zm00001eb202200	O Methyltransferase	-2.02305
Zm00001eb414190	Terpene synthase, N terminal domain (Terpene C)	-2.08717
Zm00001eb219870	Phenylalanine N monooxygenase (CYP79A2)	-2.22594
Zm00001eb020100	Flavonoid 3' monooxygenase / Flavonoid 3' hydroxylase	-2.25578
Zm00001eb155650	S adenosyl L methionine:carboxyl methyltransferase	-3.04816
Zm00001eb199390	Jasmonate O methyltransferase (E2.1.1.141)	-4.43465
2022 vs. 2020		
Zm00001eb058060	Isoflavone 2' hydroxylase / Isoflavone 2' monooxygenase	3.243446
Zm00001eb098950	Flavonol 3 O glucosyltransferase / UDP glucose flavonol 3	3.1774
Zm00001eb058070	Isoflavone 2' hydroxylase / Isoflavone 2' monooxygenase	2.720413
Zm00001eb311610	Flavanone 7 O beta glucosyltransferase	2.033281
Zm00001eb058050	Isoflavone 2' hydroxylase / Isoflavone 2' monooxygenase	2.030603
Zm00001eb058110	Isoflavone 2' hydroxylase / Isoflavone 2' monooxygenase	1.824407
Zm00001eb138390	Glucuronoxylan 4 O methyltransferase (GXM)	1.8116
Zm00001eb027940	Flavonol synthase / FLS	1.414422
Zm00001eb212480	Sterol 24 C methyltransferase (2.1.1.41, SMT1, ERG6)	1.245064
Zm00001eb427320	Neomenthol dehydrogenase / Monoterpenoid dehydrogenase	1.116041
Zm00001eb135800	Homocysteine S methyltransferase	1.081208
Zm00001eb095790	Isoflavone 7 O beta	-1.07901
Zm00001eb022840	Methyltransferase	-1.21344
Zm00001eb255340	Methyltransferase	-1.33398
Zm00001eb164860	2.4 dihydroxy 1.4 benzoxazin	-1.4653

Zm00001eb216190	Methyltransferase	-1.52463
Zm00001eb165580	2 hydroxy 1.4 benzoxazin	-1.62568
Zm00001eb428870	Aluminium activated malate transporter (ALMT)	-1.6344
Zm00001eb009600	MATE efflux family protein	-1.67645
Zm00001eb077230	Phenylalanine/tyrosine ammonia lyase (PTAL)	-1.67657
Zm00001eb394430	Isoflavonoid synthase	-1.93921

TABLE S5. *Differentially expressed genes in maize roots related to cell wall structure. Two maize genotypes, the B73 wild type (WT) and root hair deficient mutant 3 (rth3) were grown on the substrates loam and sand in three years with different precipitation levels (2020 – dry, 2021 – moist, 2022 – dry). Genes were selected from a list of differentially expressed genes comparing: 2020 vs. 2021, 2022 vs. 2021 and 2022 vs.2020. Filtering was done by searching for the following words: expansin, xyloglucan, lignin, cellulose, cellulase, pectin, trehalose, suberin. Note that the first part of the list considers differences in gene expression between the years 2020 and 2021, the second 2022 and 2021, and the third 2022 vs 2020. In 2020 n = 3 for plants grown on loam and n = 4 for plants grown in sand; in 2021 & 2022 n = 6, except for rth3 mutant grown in loam (n = 5). Positive and negative Log2fold indicates up-and downregulation, respectively.*

Gene ID	Annotation	Log2fold
2020 vs. 2021		
Zm00001eb312000	Xyloglucan: xyloglucosyl transferase	-1.13698
Zm00001eb197690	Xyloglucan 6 xylosyltransferase / Xyloglucan 6 alpha D	-1.16501
Zm00001eb170830	Xyloglucan 4 glucosyltransferase	-1.17905
Zm00001eb178230	Xyloglucan: xyloglucosyl transferase / Xyloglucan endotransglycosylase	-1.18599
Zm00001eb193680	Xyloglucan fucosyltransferase	-1.2078
Zm00001eb081440	Xyloglucan fucosyltransferase	-1.21783
Zm00001eb248500	Expansin, cellulose binding like domain	-1.2761
Zm00001eb071640	Xyloglucan: xyloglucosyl transferase / Xyloglucan endotransglycosylase	-1.3093
Zm00001eb412710	Xyloglucan 4 glucosyltransferase	-1.3573
Zm00001eb104880	Expansin/pollen allergen, DPBB domain	-1.3993
Zm00001eb117920	Xyloglucan fucosyltransferase	-1.44218
Zm00001eb391370	Expansin, cellulose binding like domain	-1.44352
Zm00001eb391450	Expansin, cellulose binding like domain	-1.45551
Zm00001eb149460	Expansin A8	-1.46712
Zm00001eb406590	Xyloglucan 6 xylosyltransferase	-1.49837
Zm00001eb299170	Xyloglucan fucosyltransferase	-1.54217
Zm00001eb014560	Xyloglucan 6 xylosyltransferase / Xyloglucan 6 alpha D	-1.57684
Zm00001eb128450	Expansin	-1.64873
Zm00001eb299020	Xyloglucan fucosyltransferase	-1.70374
Zm00001eb123560	Xyloglucan 6 xylosyltransferase	-1.79772
Zm00001eb237600	Expansin	-1.80492
Zm00001eb314930	Xyloglucan fucosyltransferase	-1.85056
Zm00001eb228410	Expansin like B1	-1.88037
Zm00001eb406700	Xyloglucan 6 xylosyltransferase	-1.94107
Zm00001eb135460	Plant invertase/pectin methylesterase inhibitor (PMEI)	-1.98137
Zm00001eb423590	Xyloglucan fucosyltransferase	-2.23158
Zm00001eb261090	Expansin	-2.25879
Zm00001eb429670	Expansin	-2.29738
Zm00001eb187650	Pectinesterase / Pectin methylesterase	-2.37236
Zm00001eb373230	Xyloglucan fucosyltransferase	-2.64589

Zm00001eb261070	Expansin	-3.06054
2022 vs. 2021		
Zm00001eb426560	Xyloglucan 6 xylosyltransferase / Xyloglucan 6 alpha D	2.263716
Zm00001eb197870	Xyloglucan endotransglucoseylase/hydrolase protein 4 related	2.079459
Zm00001eb226490	Xyloglucan endotransglucosylase/hydrolase protein 14 related	1.680805
Zm00001eb177020	Glycosyltransferase family 20 / Trehalose-phosphatase	1.519691
Zm00001eb237240	Xyloglucan: xyloglucosyl transferase	1.392346
Zm00001eb063540	Pectinesterase / Pectin methylesterase	1.339576
Zm00001eb071650	Xyloglucan: xyloglucosyl transferase	1.255454
Zm00001eb153280	Trehalose phosphate synthase 7	1.123525
Zm00001eb393070	Cellulose binding family II/chitobiase, carbohydrate binding domain	-1.01578
Zm00001eb353280	Trehalose 6 phosphatase synthase	-1.07153
Zm00001eb081440	Xyloglucan fucosyltransferase	-1.10319
Zm00001eb423590	Xyloglucan fucosyltransferase	-1.1412
Zm00001eb327190	Pectinesterase QRT1	-1.14768
Zm00001eb346520	Pectinesterase	-1.22142
Zm00001eb047650	Cellulose synthase A catalytic subunit 4	-1.2339
Zm00001eb042260	Trehalose 6 phosphate synthase	-1.37331
Zm00001eb046440	Cellulose synthase A catalytic subunit 4	-1.4117
Zm00001eb178230	Xyloglucan: xyloglucosyl transferase / Xyloglucan endotransglycosylase	-1.46207
Zm00001eb293110	Trehalose 6 phosphate synthase	-1.56916
Zm00001eb254700	Pectin methyltransferase QUA2 related	-1.62451
Zm00001eb194210	Trehalose phosphate phosphatase D related	-1.79069
Zm00001eb327900	Trehalose 6 phosphate synthase	-1.80307
Zm00001eb391400	Expansin	-1.84167
Zm00001eb339720	Pectin lyase like protein	-1.88485
Zm00001eb023420	Expansin, cellulose binding like domain	-1.90223
Zm00001eb391360	Expansin	-1.93061
Zm00001eb196090	Plant invertase/pectin methylesterase inhibitor (PMEI)	-2.162
Zm00001eb047360	Expansin	-2.18138
Zm00001eb019380	Pectinesterase / Pectin methylesterase	-2.43402
Zm00001eb000660	Xyloglucan: xyloglucosyl transferase	-2.4503
Zm00001eb047390	Expansin	-2.53534
Zm00001eb373230	Xyloglucan fucosyltransferase	-2.59674
Zm00001eb063520	Pectinesterase inhibitor domain	-3.11976
Zm00001eb261080	Expansin	-5.04632
Zm00001eb261070	Expansin	-5.19738
Zm00001eb261110	Expansin	-5.60333
Zm00001eb261000	Expansin	-5.92061
Zm00001eb261090	Expansin	-6.17605
Zm00001eb429670	Expansin	-6.46529
2022 vs. 2020		
Zm00001eb396270	Xyloglucan 6 xylosyltransferase	5.35465
Zm00001eb197870	Xyloglucan endotransglucosylase/hydrolase protein 4 related	2.516633

Zm00001eb406710	Xyloglucan 6 xylosyltransferase / Xyloglucan 6 alpha D	2.447182
Zm00001eb406700	Xyloglucan 6 xylosyltransferase	2.144797
Zm00001eb177120	Pectinesterase inhibitor 32 related	1.138526
Zm00001eb414390	Xyloglucan: xyloglucosyl transferase	-1.14994
Zm00001eb393070	Cellulose binding family II/chitobiase, carbohydrate binding domain	-1.17074
Zm00001eb042260	Trehalose 6 phosphate synthase	-1.40187
Zm00001eb353280	Trehalose 6 phosphate synthase	-1.42401
Zm00001eb293110	Trehalose 6 phosphate synthase	-1.57094
Zm00001eb327900	Trehalose 6 phosphate synthase	-1.59118
Zm00001eb346520	Pectinesterase	-1.70254
Zm00001eb196090	Plant invertase/pectin methylesterase inhibitor (PMEI)	-2.23945
Zm00001eb392520	Trehalose 6 phosphate synthase	-2.28904
Zm00001eb429670	Expansin	-4.15433
Zm00001eb261110	Expansin	-4.42797
Zm00001eb261000	Expansin	-4.61711

TABLE S6. *Maximum enzymatic rate (V_{max}) and affinity constant (K_m) values for β-glucosidase (BG), acid phosphatase (AP), leucine aminopeptidase (LAP) and N-acetylglucosaminidase (NAG) in the rhizosphere collected from B73 maize wild type (WT) and root hair deficient mutant 3 (rth3) grown on the substrates loam (L) and sand (S), in three years with different precipitation levels (2020 - dry, 2021 - moist, 2022 - dry). Grey-shaded cells represent outliers that were excluded from figure plotting. Details on the outlier filtering procedure are provided in the Materials and Methods section. $Mean_{orig}$ indicates the mean of all original (unfiltered) values, while $Mean_{filtered}$ includes only values used for figure generation (excluding outliers).*

Year	Soil-Genotype	BG		AP		LAP		NAG	
		V_{max}	K_m	V_{max}	K_m	V_{max}	K_m	V_{max}	K_m
2020	L-WT	366.65	16.39	454.73	8.19	51.6	5.03	105.84	41.92
		583.26	36.79	412.79	11.84	128.42	13.32	121.9	50.85
		454.86	33.95	397.39	10.63	133.69	16.18	79.18	23.44
		544.63	32.73	266.79	10.02	135.33	18.82	88.61	43.1
		352.51	25.83	371.72	14.08	157.84	33.9	71.38	32.46
		1146.24	152.54	369.84	11.19	179.27	31.06	133.54	89.35
	**$Mean_{orig}$**	**574.6917**	**49.705**	**378.8767**	**10.99167**	**131.025**	**19.71833**	**100.075**	**46.85333**
	**$Mean_{filtered}$**	**574.6917**	**49.705**	**401.294**	**10.92**	**146.91**	**22.656**	**100.075**	**38.354**
	L-*rth3*	1298.72	71.51	490.95	15.09	170.69	14.97	122.23	68.94
		282.4	12.89	394.75	9.19	130.62	13.56	250.44	78.51
		1051.51	51.35	587.23	18.56	153.41	23.38	114.67	35.2
		653.89	48.51	380.97	10.24	136.31	16.16	130.01	77.83
		1151.04	56.82	599.18	13.39	227.84	27.61	160.52	22.61
		744.87	42.92	447.36	15.58	175.84	37.35	120.07	42.94
	**$Mean_{orig}$**	**863.7383**	**47.33333**	**483.4067**	**13.675**	**165.785**	**22.17167**	**149.6567**	**54.33833**
	**$Mean_{filtered}$**	**560.3867**	**49.9**	**483.4067**	**13.675**	**153.374**	**22.17167**	**121.745**	**54.33833**
	S-WT	712.11	66.77	274.69	18.66	27.01	7.9	43.91	95.37
		412.8	64.25	127.71	17.12	3.74	0.72	19.55	28.69
		1379.04	63.33	365.49	18.83	22.86	4.37	45.29	55.23
		616.54	55.5	526.76	15.02	47.4	9.52	59.64	30.32
		214.13	69.86	177.69	9.59	74.25	444.72	23.6	74.65
		164.83	39.47	71.66	10.56	51.52	226.55	12.25	38.23
	**$Mean_{orig}$**	**583.2417**	**59.86333**	**257.3333**	**14.96333**	**37.79667**	**115.63**	**34.04**	**53.74833**
	**$Mean_{filtered}$**	**424.082**	**66.0525**	**257.3333**	**14.96333**	**44.608**	**5.6275**	**34.04**	**53.74833**
	S-*rth3*	583.77	79.6	217.11	21.84	85.33	25.5	22	47.66
		392.26	66.98	123.41	8.54	3.93	1.3	17.53	21.53
		975.43	74.86	307.3	14.47	36.82	7.04	40.83	29.82
		206.55	16.58	177.25	8.14	27.08	6.88	27.28	24.9

			295.79	43.87	100.66	15.02	32.06	24	22.19	33.93
			No sample	No sample	No sample	No sample	No sample	No sample	No sample	No sample
		**Mean$_{orig}$**	**490.76**	**56.378**	**185.146**	**13.602**	**37.044**	**12.944**	**25.966**	**31.568**
		**Mean$_{filtered}$**	**369.5925**	**66.3275**	**185.146**	**13.602**	**31.98667**	**12.944**	**22.25**	**27.545**
**2021**	L-WT		211.46	69.18	109.02	9.92	68.07	18.66	55.55	59.33
			133.27	21.61	108.78	8.84	66.6	13.35	59.3	71.67
			169.87	28.38	113.46	10.65	72.7	17.31	32.61	15.09
			No sample	No sample	No sample	No sample	60.24	25.4	No sample	No sample
			111.85	19.57	102.53	7.4	70.11	12.51	29.65	33.44
			244.94	36.25	151.87	10.96	94.33	17.46	82.93	105.98
		**Mean$_{orig}$**	**174.278**	**34.998**	**117.132**	**9.554**	**72.00833**	**17.44833**	**52.008**	**57.102**
		**Mean$_{filtered}$**	**174.278**	**26.4525**	**117.132**	**9.554**	**67.544**	**15.858**	**52.008**	**57.102**
	L-*rth3*		126.18	25.54	114.25	11.02	50.12	15.38	70	185.44
			264.37	45.16	148.78	11.13	76.31	16.65	274.07	604.43
			250.84	41.59	138.87	10.11	105.45	21.25	42.55	38.29
			231.4	49.83	103.61	8.08	74.84	18.46	93.75	207.93
			276.14	42.78	132.98	10.42	66.73	14.26	40.25	58.44
			87.36	10.46	95.67	6.11	60.25	13.89	43.38	54.37
		**Mean$_{orig}$**	**206.0483**	**35.89333**	**122.36**	**9.478333**	**72.28333**	**16.64833**	**94**	**191.4833**
		**Mean$_{filtered}$**	**206.0483**	**35.89333**	**122.36**	**9.478333**	**65.65**	**16.64833**	**42.06**	**50.36667**
	S-WT		58.5	16.27	61.82	5.58	26.9	15.2	28.69	55.19
			56.71	22.72	31.52	3.25	21.22	11.43	10.86	30.43
			13.21	11.41	19.79	11.76	10.19	35.96	4.13	46.04
			159.48	70.35	40.03	9.12	21.05	18.3	10.94	32.94
			24.02	17.39	17.58	6.53	6.43	13.14	5.34	30.83
			27.05	19.27	19.99	5.69	4.93	4.99	5.5	28.52
		**Mean$_{orig}$**	**56.495**	**26.235**	**31.78833**	**6.988333**	**15.12**	**16.50333**	**10.91**	**37.325**
		**Mean$_{filtered}$**	**35.898**	**17.412**	**31.78833**	**6.988333**	**15.12**	**14.5175**	**7.354**	**37.325**
	S-*rth3*		107.51	70.22	34.33	8.61	78.76	70.53	20.91	32.49
			41.98	29.85	32.62	7.67	31.29	32.51	22.88	48
			53.45	52.38	16.93	7.08	27.12	43.04	12.98	22.99
			153.37	78.66	20.86	8.76	11.88	19.58	6.5	22.63
			37.84	37.39	15.92	7.94	8.98	21.36	4.69	60.99
			1525.84	92.59	174.95	26.59	17.04	19.82	18.2	39.36
		**Mean$_{orig}$**	**319.9983**	**60.18167**	**49.26833**	**11.10833**	**29.17833**	**34.47333**	**14.36**	**37.74333**
		**Mean$_{filtered}$**	**78.83**	**53.7**	**24.132**	**8.012**	**19.262**	**20.25333**	**14.36**	**37.74333**
**2022**	L-WT		212.38	13.24	244	15.35	209.54	49.25	107.29	25.74
			217.88	24.97	230.55	15.28	137.48	27.05	58.96	15.59
			352.84	22.52	327.32	34.28	169.17	24.08	116.43	22.68
			186.58	29.49	138.46	9.5	99.72	30.85	55.25	22.17

	265.89	32.08	274.06	32.54	125.26	17.72	63.87	13.59
	406.02	41.39	239.82	16.32	133.39	20.78	61.18	32.26
**Mean$_{orig}$**	**273.5983**	**27.28167**	**242.3683**	**20.545**	**145.76**	**28.28833**	**77.16333**	**22.005**
**Mean$_{filtered}$**	**247.114**	**24.46**	**236.03**	**14.1125**	**133.004**	**24.096**	**77.16333**	**22.005**
	223.04	22.1	190.7	11.08	136.84	30.02	61.88	27.28
	469.48	97.79	204.78	17.71	133.27	23.73	60.29	24.54
L-*rth3*	255.66	24.98	232.34	22.73	131.97	30.62	66.91	23.14
	412.03	49.32	200.23	11.33	133.74	33.75	66.14	70.43
	451.46	45.05	213.2	13.97	109.41	19.98	53.53	23.71
	179.61	16.53	150.53	6.56	103.58	23.3	44.26	21.41
**Mean$_{orig}$**	**331.88**	**42.62833**	**198.63**	**13.89667**	**124.8017**	**26.9**	**58.835**	**31.75167**
**Mean$_{filtered}$**	**304.36**	**31.596**	**198.63**	**13.89667**	**124.8017**	**26.9**	**58.835**	**24.016**
	99.73	17.58	127.45	9.08	62.02	24.29	42.78	18.35
	157.6	21.43	122.55	9.61	82.02	25.02	34.01	10.9
S-WT	148.1	31.75	141.43	20.05	69.53	37.39	47.83	28.93
	144.04	45.72	138.25	18.19	62.3	34.07	52.97	38.16
	68.16	24.86	104.05	18.41	37.34	23	22.57	19.26
	48.96	13.36	103.25	47.35	24.54	30.24	26.51	59.98
**Mean$_{orig}$**	**111.0983**	**25.78333**	**122.83**	**20.44833**	**56.29167**	**29.00167**	**37.77833**	**29.26333**
**Mean$_{filtered}$**	**111.0983**	**25.78333**	**132.42**	**14.2325**	**56.29167**	**29.00167**	**37.77833**	**23.12**
	89.39	12.4	111.2	7.45	49.26	27.18	34.32	22.07
	113.79	26.86	106.15	10.76	39.77	13.42	26.69	23.92
S-*rth3*	81.36	15.19	130.09	16.46	34.83	9.65	36.28	30.42
	72.36	16.84	95.09	20.4	40.35	18.97	30.17	30.28
	88.22	18.91	154.33	53.7	54.86	38.56	31.87	17.47
	109.51	78.07	59.09	10.42	23.42	26	20.44	30.44
**Mean$_{orig}$**	**92.43833**	**28.045**	**109.325**	**19.865**	**40.415**	**22.29667**	**29.96167**	**25.76667**
**Mean$_{filtered}$**	**92.43833**	**18.04**	**100.324**	**13.098**	**40.415**	**22.29667**	**29.96167**	**25.76667**

TABLE S7. *Nutrient concentrations of calcium (Ca), potassium (K), magnesium (Mg), phosphorus (P) and nitrogen (N) in the youngest, unfolded leaf of B73 maize wild type (WT) and root hair deficient mutant 3 (rth3) plants grown on the substrates loam (L) and sand (S). Samples were taken during two dry years (2020 and 20222) at the four-leaf stage (BBCH14). Kruskal-Wallis Test followed by Dunn's Test was used to display significance levels between substrates: *p< 0.001, **p < 0.01, ***p < 0.001.*

**Treatment**	**2020** Ca [mg/g]	**2022** Ca [mg/g]	**2020** K [mg/g]	**2022** K [mg/g]	**2020** Mg [mg/g]	**2022** Mg [mg/g]	**2020** P [mg/g]	**2022** P [mg/g]	**2020** N [mg/g]	**2022** N [mg/g]
**L-WT**	7.94	10.94	8.28	5.34	6.72	6.67	2.75	4.13	26.65	27.24
**L-*rth3***	8.50	11.38	6.82	5.56	7.19	6.81	3.29	4.51	30.24	29.66
**S-WT**	6.15	10.53	17.58	9.54	4.83	5.35	2.10	2.82	28.66	19.71
**S-*rth3***	7.08	10.18	16.82	11.23	4.77	4.74	2.23	2.73	30.16	21.87
**Significances between Substrates**	**	n.s.	***	***	***	***	***	***	n.s.	***
**Loam: Significances between Years**	***		**		n.s.		***		n.s.	
**Sand: Significances between Years**	***		***		n.s.		*		***	

Table S8. *Genes associated with the rth3 (root hair defective 3) mutation examined in comparisons between B73 maize wild type (WT) and rth3 mutant roots. Plants were grown on the substrates loam and sand in three years with different precipitation levels (2020 – dry, 2021 – moist, 2022 – dry). In 2020 n = 3 for plants grown on loam and n = 4 for plants grown in sand; in 2021 & 2022 n = 6, except for rth3 mutant grown in loam (n = 5). Positive and negative Log2fold indicates up-and downregulation, respectively. Note that genes are considered to be differentially expressed with absolute Log2fold > 1.*

**Gene ID**	**Annotation**	**Log2fold**	
		**Loam**	**Sand**
2020			
Zm00001eb014120	COBRA-like protein 7 (Roothairless 3)	-1.216687	-3.151493
Zm00001eb031250	Protein root hair defective 3 homolog 1	-0.016296	0.077287
Zm00001eb135700	Protein root hair defective 3 homolog 1	-0.499691	-0.979744
2021			
Zm00001eb014120	COBRA-like protein 7 (Roothairless 3)	-3.235767	-3.008841
Zm00001eb031250	Protein root hair defective 3 homolog 1	-0.177213	0.487001
Zm00001eb135700	Protein root hair defective 3 homolog 1	0.251567	0.804017
2022			
Zm00001eb014120	COBRA-like protein 7 (Roothairless 3)	-2.535644	-2.652398
Zm00001eb031250	Protein root hair defective 3 homolog 1	0.088638	0.061001
Zm00001eb135700	Protein root hair defective 3 homolog 1	-0.546468	-0.370608

TABLE S9. *Permutational analysis of variance (PERMANOVA) of root gene expression levels. Samples were taken at three years with different precipitation (2020 – dry, 2021 – moist, 2022 – dry) from two maize genotypes, B73 wild type and root hair deficient mutant 3 (rth3) grown on the substrates loam and sand. 'Adonis' function was used to display R^2 and significance levels: **p < 0.01, ***p < 0.001.*

	**Loam**			**Sand**		
	2020 & 2021	2022& 2021	2022 & 2020	2020 & 2021	2022 & 2021	2022 & 2020
	R^2	R^2	R^2	R^2	R^2	R^2
Year	0.24***	0.32***	0.27***	0.21***	0.44***	0.38***
Genotype	0.05	0.03	0.03	0.05	0.03	0.05
Year: Genotype	0.03	0.02	0.04	0.04	0.02	0.02
Residuals	0.68	0.63	0.66	0.7	0.5	0.54
